# Structural and health system determinants of mental health in Tanzania: mapping policy recommendations to the WHO comprehensive mental health action plan 2013–2030

**DOI:** 10.3389/fpubh.2026.1878653

**Published:** 2026-07-10

**Authors:** Monika Sawhney, Ana Carla Soares Portugal Schippert, Mercy Chiduo, Angelina M. Lutambi, Basiliana Emidi, Olav Johannes Hovland, Ingjerd Legreid Ødemark, Camilla Thorvik, Ellen Karine Grov, Sezer Kisa

**Affiliations:** 1 Department of Public Health, Marshall University, Huntington, WV, United States; 2 Department of Nursing and Health Promotion, Faculty of Health Sciences, Oslo Metropolitan University, Oslo, Norway; 3 National Institute for Medical Research, Dar es Salaam, Tanzania; 4 Library, Oslo Metropolitan University, Oslo, Norway

**Keywords:** health policy, mental health, scoping review, structural determinants, Tanzania, WHO framework

## Abstract

**Background:**

Mental health disorders are a major public health concern in Tanzania, where most people in need of care do not receive treatment and mental health services remain severely under-resourced. Despite a growing body of country-level research, no prior review has mapped the evidence on structural determinants of mental health conditions and policy recommendations within the WHO Comprehensive Mental Health Action Plan 2013–2030.

**Objectives:**

This scoping review aimed to identify structural and health system factors associated with mental health conditions in Tanzania and map policy recommendations reported in the included studies against the objectives of the WHO Comprehensive Mental Health Action Plan 2013–2030.

**Methods:**

Following the Joanna Briggs Institute framework for scoping reviews, five electronic databases (Embase, MEDLINE, PsycINFO, Web of Science, and Scopus) were searched for peer-reviewed empirical studies published in English up to June 2025. Screening and full-text assessment were conducted independently by multiple reviewers using Covidence. Data were extracted using a standardized extraction form and synthesized through deductive thematic analysis organized within the domains of the WHO Comprehensive Mental Health Plan.

**Results:**

In this review, 74 studies met the inclusion criteria. Depression and anxiety were the most frequently studied mental health conditions. The main structural and health system factors associated with mental health conditions were poverty, food insecurity, and health-system weaknesses. Policy recommendations primarily emphasized service integration and community-level promotion, whereas governance reform, sustainable financing, and culturally adapted measurement tools were less frequently addressed.

**Conclusions:**

The findings highlight persistent gaps in leadership, governance, financing, and mental health information systems. Addressing these gaps requires coordinated, system-oriented strategies aligned with all levels of the WHO framework, particularly stronger attention to governance and financing, which were less frequently addressed in the recommendations from the included studies.

## 1 Introduction

Mental health conditions are a significant global public health problem (1). According to the World Health Organization (WHO), more than one billion people live with mental health conditions, and mental, neurological, and substance use disorders collectively account for approximately 13% of the global burden of disease (2). Depression and anxiety disorders, the most common forms of mental ill-health, affect millions of people globally and cause substantial suffering, reduced quality of life, impaired social functioning, and major economic losses (3–5). People living with mental disorders have a life expectancy that is up to 20 years shorter than that of the general population (6, 7). Despite this burden, mental health has received limited attention in global health policy, research, and resource allocation. Critically, this neglect is most acute in low- and middle-income countries, where the majority of affected individuals reside yet where research, policy, and investment remain most limited (8).

Sub-Saharan Africa (SSA) faces a particularly heavy burden of mental ill-health, shaped by economic insecurity, rapid urbanization, fragile health systems, and longstanding underinvestment in mental healthcare (3, 8, 9). According to WHO, nearly 150 million people in Africa are living with mental health conditions, with limited access to decentralized and integrated care, highlighting the region's mental health challenges (10). As Sankoh and colleagues highlighted, the scarcity of mental health research in Africa mirrors the weakness of mental health services across the continent and the limited political will to address the problem (8). The biomedical approach in global health governance is sustained by donor funding, international health institutions, and policy frameworks, which marginalize mental health and locally grounded care. Service gaps therefore reflect not only resource deficits but also political and governance conditions (11).

Tanzania is one of several countries in SSA where these challenges are particularly evident. Although the country has made some progress in expanding its mental health workforce, service capacity remains critically limited. Recent estimates suggest that Tanzania has approximately 38 psychiatrists, 495 mental health nurses, 17 psychologists, and 29 social workers for a population of more than 65 million, equivalent to 1.31 mental health workers per 100,000 population, far below recommended thresholds (7). By comparison, the global average stands at 13.5 mental health workers per 100,000 population, while Africa averages 2.2 per 100,000 (12). Mental health services remain concentrated in urban tertiary settings (13), while essential psychotropic medicines are often unavailable at primary care level (14, 15). Community-based mental healthcare is still underdeveloped, and task-shifting initiatives have not been implemented consistently across settings (16, 17).

Although global mental health research and policy have expanded considerably over the past two decades, including through the WHO Comprehensive Mental Health Action Plan 2013–2030, evidence remains unevenly translated into contextually relevant strategies in many SSA settings (8, 11, 18). Commitments to expand severe mental disorder service coverage have largely not been met across Africa, revealing a major implementation gap (19). In Tanzania, this gap reflects contested public authority in mental health, where biomedical, traditional, faith-based, and community actors shape access to care without strong coordination or referral systems (11, 20). Both systems also tend to focus on individual treatment while giving less attention to the social determinants that sustain poor mental health, including poverty, food insecurity, violence, and unemployment (11, 21, 22).

Although previous reviews have examined mental health in sub-Saharan Africa or focused on specific Tanzanian populations, these broader or population-specific syntheses are insufficient for informing Tanzania's national mental health policy and implementation priorities (3, 23, 24). Regional reviews often aggregate evidence across countries with different health system structures, financing arrangements, service delivery models, cultural pathways to care, and policy environments. As a result, they may obscure country-specific determinants and implementation barriers that are essential for national planning. Tanzania has a distinctive mental health context, including severe workforce shortages, urban concentration of specialist services, limited availability of psychotropic medicines at primary care level, weak community-based follow-up, and pluralistic care-seeking involving biomedical, traditional, faith-based, and community actors. These conditions require a Tanzania-specific synthesis that can identify how structural determinants and health system constraints are reflected in the national evidence base.

This review is informed by a system-oriented perspective in which mental health outcomes are shaped by both structural determinants and health system factors. In this review, structural determinants refer to the wider social, economic, and environmental conditions that influence mental health risk and vulnerability, including poverty, unemployment, food insecurity, education, gendered disadvantage, violence, and social exclusion. Health system determinants refer to features of service organization and delivery that influence access to, quality of, and continuity of mental healthcare, including workforce capacity, service accessibility, medication availability, screening practices, referral pathways, follow-up care, and integration of mental health into primary and community-based services (21, 25). Although these factors are conceptually distinct, they are closely connected: structural disadvantage can increase the need for mental health support, while health-system weaknesses can limit timely access to care and worsen unmet need. Policy recommendations were therefore interpreted as responses to structural determinants, health system constraints, or both. When mapped to the WHO Comprehensive Mental Health Action Plan 2013–2030, structural determinants were most closely linked to recommendations on leadership and governance, promotion and prevention, and intersectoral action, whereas health system factors were most closely linked to service delivery, workforce development, access and quality of care, medication availability, and information systems, evidence, and research (26). This framing allowed the review to distinguish social and structural conditions from health system constraints while examining how both were reflected in the Tanzania-specific evidence base.

The review addresses the following questions:

1. What structural and health system-level factors have been reported in relation to mental health conditions in Tanzania?
2. What policy recommendations have been proposed to strengthen the mental health system in Tanzania, and how do these align with the WHO Comprehensive Mental Health Action Plan 2013–2030?

## 2 Method

This review was designed as a scoping review to map and synthesize evidence on the multilevel determinants of mental health conditions in Tanzania by following the methodological framework outlined by the Joanna Briggs Institute for scoping reviews (27). The review team was established through a collaboration between researchers from Tanzania and Norway, including two research librarians. An initial scoping search using the broad terms “mental health” and “Tanzania,” conducted by one of the librarians, confirmed the existence of prior review studies on the topic and supported the selection of a scoping review methodology as the most appropriate approach for mapping the available evidence. Early and ongoing consultation between the research librarians and the researchers informed the development of more focused and targeted research questions aligned with the review's objectives. This scoping review was not registered in a formal review registry. The review is reported in accordance with the PRISMA extension for scoping reviews (28). The completed preferred reporting items for systematic reviews and meta-analyses extension for scoping reviews (PRISMA-ScR) checklist is provided as Supplementary file 5.

### 2.1 Eligibility criteria

This review included empirical studies published in peer-reviewed journals addressing mental health conditions among populations in Tanzania, including Zanzibar. To be eligible, studies were required to report on at least one of the following: (1) structural and health system level determinants associated with common mental disorders; and (2) findings with implications for mental health practice, policy, or research in low- and middle-income country (LMIC) contexts. Eligible studies included participants of any age from the general population or key subgroups such as women, youth, older population, rural communities, and people living with chronic conditions, provided that they were affected by, or at risk of, mental health conditions. No restrictions were placed on study design, and both quantitative and qualitative studies were eligible for inclusion. Only studies published in English and available up to June 2025 were considered. The search did not restrict studies by publication year.

Studies were excluded if they did not address structural and health service-related determinants and/or policy recommendations, did not involve populations in Tanzania or Zanzibar, or were not published in peer-reviewed journals. All inclusion and exclusion criteria were entered and managed in Covidence (Clarivate Analytics, PA, USA).

### 2.2 Information sources

A comprehensive search strategy was developed by two research librarians at Oslo Metropolitan University. The search was conducted across five electronic databases: Embase, MEDLINE, PsycINFO, Web of Science, and Scopus. These five databases were selected due to their broad and complementary coverage of the literature relevant to this review. MEDLINE and Embase were included to capture biomedical, clinical, and public health research; PsycINFO was included to capture psychology, psychiatry, and mental health literature; and Scopus and Web of Science were included to provide broad multidisciplinary and citation-based coverage across health, social science, and policy-related research. Full search history is provided in Supplementary file 4.

### 2.3 Search

The search strategy consisted of two main components combined with the Boolean operator AND: mental health conditions and Tanzania. The mental health component included both specific disorders and broader terms related to mental illness and mental healthcare. Where available, controlled vocabulary terms were used and expanded, alongside free-text searches in titles, abstracts, and keywords. The search terms were cross-referenced with the International Classification of Diseases (ICD-10/ICD-11) and developed in consultation with Tanzanian collaborators to ensure contextual relevance. The geographic component included Tanzania, Zanzibar, major cities and regions with referral hospitals, and relevant hospital names when necessary. In medical and psychological databases, we searched across broader fields, including author affiliations, whereas in multidisciplinary databases, the search was limited to titles, abstracts, and keywords. The National Institute for Medical Research was not included as a standalone term because it is used in multiple countries and would not add specificity beyond the Tanzania filter.

### 2.4 Selection of sources of evidence

**Figure 1:**
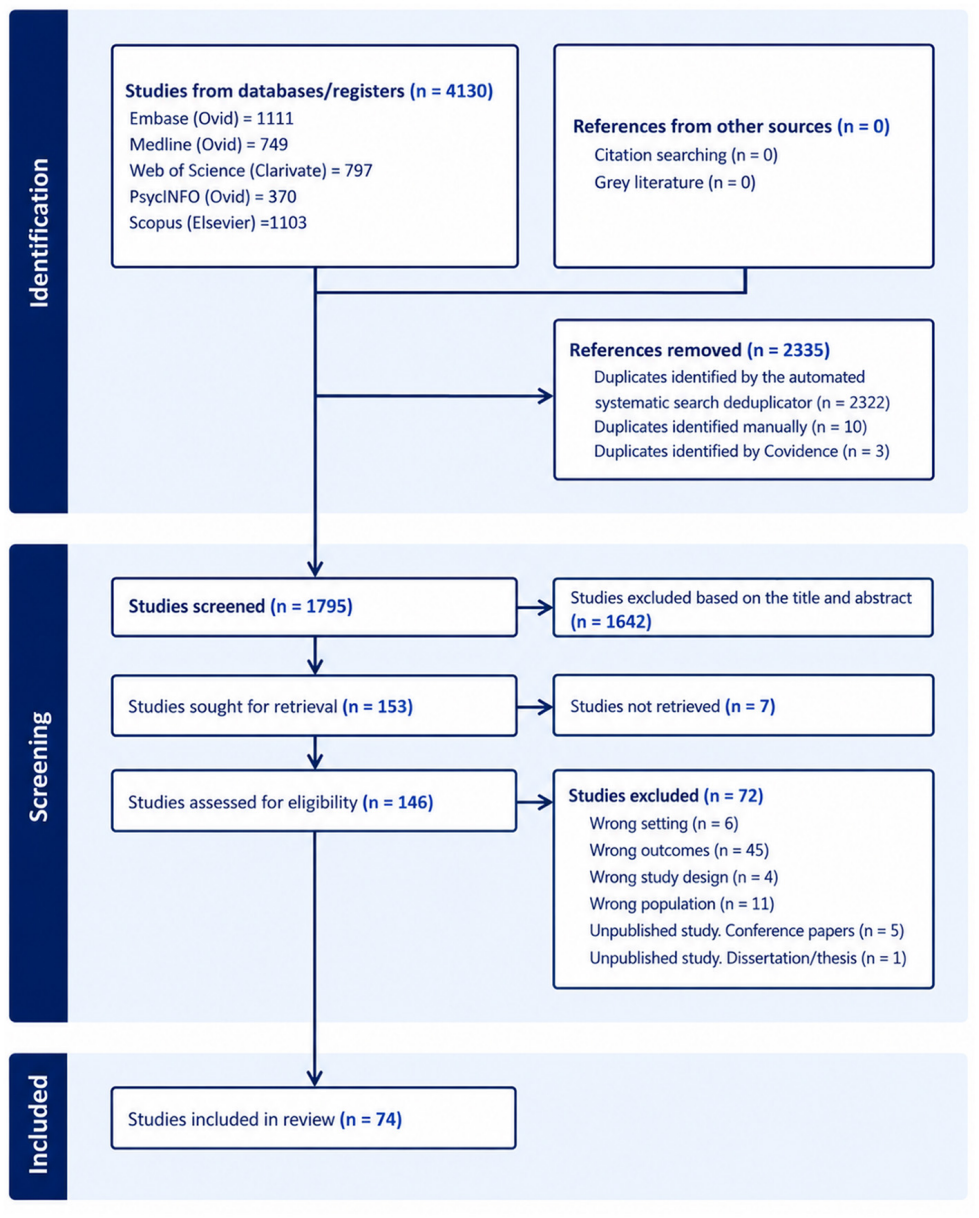
PRISMA flow diagram.

Following the database searches, all identified citations were collated and uploaded into EndNote and Covidence (Clarivate Analytics, PA, USA). Duplicate removal was conducted using the Automated Systematic Search De-duplicator (ASySD) (29), followed by additional manual deduplication by the librarians and further automated removal of records in Covidence. A total of 2,335 duplicate records were removed. Titles and abstracts were independently screened by five reviewers against the inclusion criteria. Screening conflicts at the title-and-abstract stage were resolved through discussion among the reviewers, and unresolved disagreements were discussed with the wider review team. Full texts of potentially eligible citations were assessed in detail by eight independent reviewers in Covidence, which allowed reviewers' screening decisions to remain blinded until conflicts were resolved. Disagreements at each stage were resolved through team discussion after the completion of full-text screening. A total of 74 studies met the inclusion criteria and were included in the review. The search and inclusion process, including reasons for exclusion at the full-text review stage, is presented in the PRISMA flow diagram (Figure 1).

### 2.5 Data extraction

A data extraction form was developed by two researchers and piloted with five studies to ensure consistency, clarity, and relevance prior to full data extraction. The form was revised based on feedback from the research team before data extraction. The extraction form included study characteristics (author(s), year, country, study design, setting, and population/sample), as well as findings relevant to the two research questions guiding this review. We extracted policy recommendations when the original study authors explicitly stated recommendations, policy or practice implications, or proposed actions to improve mental health services, prevention, research, or health system responses in Tanzania. The review team did not develop new recommendations during extraction but recorded and organized the recommendations and policy-relevant implications reported in the included studies.

### 2.6 Critical appraisal of individual sources of evidence

As this study was designed as a scoping review to map the existing literature, the included studies were not assessed for quality. This is consistent with methodological guidance for scoping reviews, which does not require critical appraisal of individual sources of evidence as a standard component of the review process (30).

### 2.7 Synthesis of results

Extracted data were organized using a standardized extraction form developed in Microsoft Excel. A deductive thematic analysis approach was used to synthesize findings across the included studies. The analysis was guided by the two research questions, which informed how the data were organized and interpreted. Extracted determinants were coded into structural and health system categories, including economic insecurity, food insecurity, unemployment, workforce shortages, medication availability, access barriers, screening and diagnostic capacity, and service integration. Policy recommendations were extracted when the original study authors explicitly reported recommendations, policy or practice implications, or proposed actions for improving mental health services, prevention, research, or health system responses in Tanzania. The review team did not create new policy recommendations; rather, author-reported recommendations were coded, organized, and synthesized. Recommendations were mapped deductively to the four objectives of the WHO Comprehensive Mental Health Action Plan 2013–2030: leadership and governance; comprehensive, integrated, and responsive mental health and social care services; promotion and prevention; and information systems, evidence, and research. To provide a more detailed synthesis, recommendations that were first mapped to the WHO Action Plan objectives were further grouped into seven analytic domains. These domains were developed through an iterative coding process that combined the WHO framework with recurring recommendation themes identified across the included studies. Recommendations addressing policy direction, coordination, accountability, or financing were grouped under leadership and governance; those focused on integrating mental health into existing health services or expanding care platforms were grouped under service integration and expansion; recommendations on training, task-sharing, or provider capacity were grouped under workforce development; recommendations on affordability, geographic access, referral pathways, follow-up care, and quality of care were grouped under access and service quality; recommendations concerning psychotropic medicine supply were grouped under medication availability; recommendations on stigma reduction, screening, early identification, and prevention were grouped under promotion and prevention; and recommendations on research, culturally adapted tools, data systems, and evidence generation were grouped under information systems, evidence, and research. When a recommendation could reasonably fit more than one domain, it was assigned to the domain that reflected its main policy focus after discussion between two reviewers (MS and SK).

## 3 Results

### 3.1 Selection of sources of evidence

In total, 4,130 records were identified. After removing 2,335 duplicates using automated and manual procedures, 1,795 studies were screened by title and abstract. Of these, 153 articles were sought for full-text review, and 146 were assessed for eligibility. 72 studies were excluded at the full-text stage due to wrong setting, outcomes, study design, population, or because they were unpublished conference papers or dissertations. Ultimately, 74 studies met the inclusion criteria and were included in the review (Figure 1) (14, 20, 31–102). Of these, 70 reported structural or health system determinants, and 44 included policy recommendations that could be mapped to the WHO framework.

### 3.2 Characteristics of sources of evidence

Almost half of the included studies were published between 2020 and 2025 (*n* = 35), and 28.3% (*n* = 21) were published between 2015 and 2019. Most of the studies were designed as quantitative and primarily cross-sectional studies. Most of the studies were conducted across multiple regions of Tanzania, including Dar es Salaam, Kilimanjaro (Moshi, Hai), Mwanza, Zanzibar, and Arusha (Monduli). Sample sizes varied from 10 participants (86) to over 10,000 (39). Study populations were diverse, with most studies focusing on adults (Supplementary Table 1).

### 3.3 Synthesis of results

#### 3.3.1 Structural and health system factors associated with mental health outcomes

**Figure 2:**
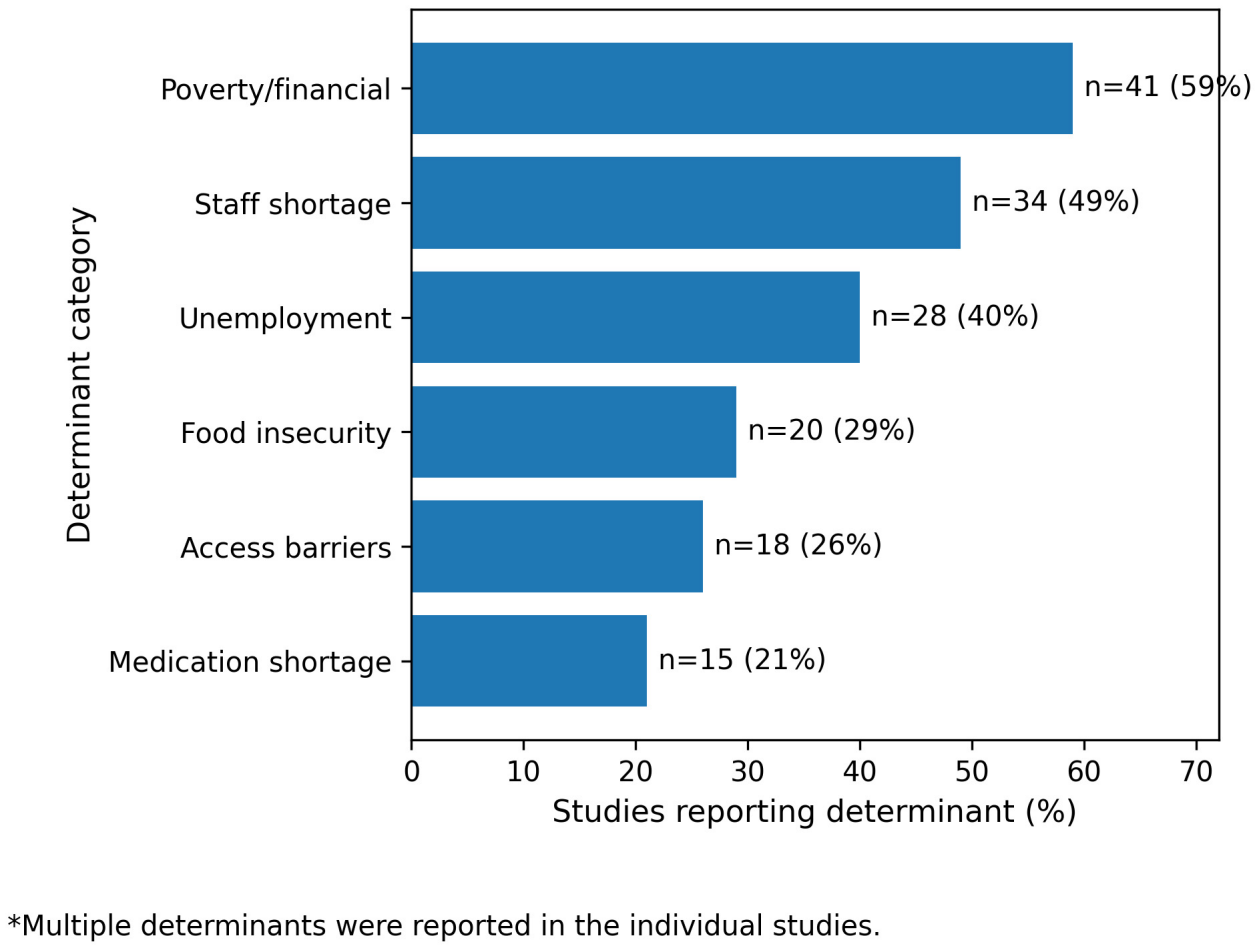
Distribution of structural and health system determinants of mental health conditions* (*n* = 70).

Under structural factors, economic factors emerged as dominant determinants across studies. Among the 70 studies that reported structural or health system determinants, poverty and financial barriers were the most commonly cited factors, appearing in 59% of studies, followed by shortage of trained mental health staff (49%) and unemployment (40%) (Figure 2), affecting diverse populations including children, older population, pregnant women, and those with chronic mental illness (41–43, 46, 57, 81, 86–88, 90–92, 100). Economic factors accounted for the largest share of all identified factors (38%), underscoring the role of socioeconomic determinants in mental health outcomes in Tanzania (Figure 2). High out-of-pocket costs, limited insurance coverage, and geographic distance to mental health services further deepened these disadvantages, particularly for rural populations (31, 38, 42–44, 92–94, 98) (Table 1).

**Table 1:** Structural and health system factors associated with mental health conditions (*n* = 70).

References/region	Mental health conditions	Structural & health system factors
Adams et al., (31)/Kilimanjaro, Tanzania	Geriatric depressive symptoms Cognitive problem	Shortage and misplacement of trained mental health professionals Frequent stock-outs of essential psychiatric medications Limited insurance coverage and high out-of-pocket costs Long travel distances to services, particularly for rural populations Low prioritization of mental health in national health planning
Alemu et al., (32)/	Depression Post-traumatic stress disorder (PTSD)	Unemployment Care stops at hospital discharge; no structured follow-up for mental health Measurement tools not fully culturally adapted — risk of under-detection Limited trained staff
Aloyce et al., (33)/	Depression Suicidal thoughts	Food insecurity Unemployment Poverty Low availability of trained professionals Limited tailored mental health services for young men Low utilization
Ambikile & Iseselo, (36)/Dar es Salaam, Tanzania	Schizophrenia Depression Bipolar disorder	Limited staff, infrastructure, funding, and medication
Ambikile, (34)/	Schizophrenia Bipolar disorder	Weak health system support Resource constraints
Ambikile, (35)/Dar es Salaam, Tanzania	Autism spectrum disorder ADHD Epilepsy/seizure disorders Mental retardation Learning disabilities	Poverty Lack of special schools Drug shortages Long waiting time at hospital
Bintabara et al., (37)/Dar es Salaam, Dodoma, Mbeya, Morogoro, and Mwanza, Tanzania	Depression Anxiety Stress	Studying in private universities Higher tuition fees Lack of mental health services in universities
Blixen, (38)/Dar es Salaam, Tanzania	Chronic psychotic disorders (schizophrenia, schizoaffective)	Limited access to medications Cost of medications Transport barriers Workforce limitations Bureaucratic barriers
Bondestam et al., (39)/Zanzibar, Tanzania	Epilepsy Chronic psychosis (including schizophrenia) Acute psychotic episodes Mental retardation Other brain-damage-related disorders	Poor access to mental health care in primary health facilities Low resources and weak health system capacity
Cherewick et al., (40)/Dar es Salaam, Tanzania	Depression Anxiety Externalizing behaviors (disobedience, aggression, substance use)	Lack of supportive resources
Daniel et al., (41)/Arusha, Tanzania	Psychosis Stress-related disorders	Limited access to health facilities in rural areas Poverty Few professionals trained in mental health care Limited availability of essential mental health treatments
Decaro et al., (42)/Mwanza, Tanzania	Depression Anxiety	Severe food insecurity Low household wealth Household adversity
Dorsey et al., (43)/Kilimanjaro, Tanzania	Post-traumatic stress (PTS) Depression Anxiety Grief Stress	Poverty (lack of food, clothing, school supplies, basic rights) Stressful living conditions (overwork, poor care, unstable living environments)
Herlosky et al., (44)/Mara, Tanzania	Postpartum depression	Distance to health facilities
Hill et al., (45) /Dar es Salaam, Tanzania	Anxiety Depression	Financial constraints
Hovland et al., (20)/Tanga, Tanzania	Caregivers' understanding of mental illness and their experience in caring for mentally ill individuals	Limited access to resources Lack of trained personnel
Howorth et al., (46)/Kilimanjaro, Tanzania	Depression	Availability of pensions and social assistance Hardship resulting from lack of economic productivity Shortage of medication
Iseselo et al., (49)/Dar es Salaam, Tanzania	Schizophrenia Major depression Bipolar disorders	Safety and protection
Iseselo et al., (48)/Dar es Salaam, Tanzania	Schizophrenia Major depression Bipolar disorders	Financial shortage of medication Inadequate availability of psychotropic medication Inadequate affordability of psychotropic medication
Iseselo et al., (47)/Dar es Salaam, Tanzania	Mental illnesses	Lack of follow-up for families and patients Financial
Ivanova et al., (50)/Mbeya and Songwe, Tanzania	Mental wellbeing Psychological distress	Financial
Jenkins et al., (52)/Dar es Salaam, Tanzania	Psychotic disease	Economic costs
Jenkins et al., (51)/Dar es Salaam, Tanzania	Depression Anxiety	Income instability Unemployment Legal issues
Jeong et al., (53)/Mwanza, Tanzania	Parenting stress, depression Anxiety Suicidal ideation Extreme sadness or loneliness	Poverty/unemployment Financial insecurity
Kaaya et al., (54)/Dar es Salaam, Tanzania	Antenatal depression	Engagement in cash earning Low/moderate satisfaction with ability to access basic needs
Khan et al., (55)/Dar es Salaam, Tanzania	Depression Suicidal ideation	Unemployment Lack of systematic depression screening in diabetes care High patient load
Knettel, (56)/Arusha and Kilimanjaro, Tanzania	Mental health illness	Scarcity of mental health providers
Knettel et al., (57)/Arusha and Kilimanjaro, Tanzania	Alcohol and substance use disorders Schizophrenia	Poverty Unemployment Limited education Shortage of trained providers Lack of knowledge about treatments available
Kuringe et al., (58)/Shinyanga, Tanzania	Depression Anxiety	Having savings Unemployment Absence of validated screening tools Limited mental health service accessibility Lack of policies and implementation plans for mental health
Lee et al., (59)/Dar es Salaam, Tanzania	Depression Anxiety	Low socioeconomic resources Lack of validated local screening tools
Lugata et al., (60)/Kilimanjaro, Tanzania	Depression Suicidal ideation Eating disorders	Lack of structured student support services
Magnusson et al., (61)/Kilimanjaro, Tanzania	Prenatal depression	Underreporting IPV and/or depression
Mahenge et al., (63)/Dar es Salaam, Tanzania	Post-traumatic stress disorder Anxiety Depression	Lack of screening integration in antenatal clinics Resource constraints Limited awareness on the issue of intimate partner violence during pregnancy
Mahenge et al., (64)/Dar es Salaam, Tanzania	Depression Anxiety Post-traumatic stress disorder	Lack of integrated screening Absence of validated PTSD tools Limited awareness among health workers
Massae et al., (67)/Pwani, Tanzania	Depressive symptoms during pregnancy	Not having a formal education Inadequate income
Massae et al., (66)/Pwani, Tanzania	Depressive symptoms	More than 12 hours at a health facility before childbirth
Mbarak et al., (68)/Dar es Salaam, Tanzania	Postpartum depression	Education
Mbatia et al., (14)/Dar es Salaam, Tanzania	Depression	Shortage of medication
Mboya et al., (69)/Kilimanjaro, Tanzania	Mental distress	Area of residence
Mbwilo et al., (70)/Dar es Salaam, Tanzania	Mental disability	Poor knowledge of availability of resources Limited access to quality health care/inadequate health care services
Mirza et al., (72)/Pemba, Zanzibar, Tanzania	Common mental disorders (CMD)/non-psychotic mental disorders [Epilepsy]	Primary health care (PHC) setting for help-seeking
Mlaki et al., (73)/Kilimanjaro, Tanzania	Depression	Lack of trained staff
Moledina et al., (74)/Dar es Salaam, Tanzania	Depression	Low income
Munisi et al., (75)/Mwanza, Tanzania	Depression	Limited mental health screening Shortage of counselors/psychiatrists Financial burden
Mussa et al., (76)/Zanzibar, Tanzania	Depression	Lack of integrated mental health screening in diabetic care Lack of routine check-ups Lack of mental health professionals in health facilities
Mwita et al., (77)/Mwanza, Tanzania	Antepartum depression	Low education College education (protective factor)
Mwita et al., (79)/Mwanza, Tanzania	Postpartum depression Generalized anxiety disorder	Monthly income
Mwita et al., (78)/Mwanza, Tanzania	Generalized anxiety disorder	Low education College education (protective) Low income Higher income (protective)
Myers et al., (80)/Arusha, Tanzania	Auditory verbal hallucinations Voice-hearing Psychological distress	Natural disasters (drought, flooding) Food insecurity Water insecurity Scarce access to care Limited health worker presence
Ndosi et al., (82)/Dar es Salaam, Tanzania	Puerperal psychosis, schizophrenia	Unemployment Poverty Overcrowded wards Limited specialists
Ndosi et al., (81)/Dar es Salaam, Tanzania	Attempted suicide Deliberate self-harm and associated psychiatric disorders	Poverty Unemployment Low education
Ngocho et al., (83)/Kilimanjaro, Tanzania	Antenatal depression and suicidal ideation	Limited screening protocols Under-resourced antenatal care system
Ngoma et al., (84) /Dar es Salaam, Tanzania	Common mental health problems	Inaccessibility of services Time limit in primary health clinics
Njiro et al., (85)/Dar es Salaam, Tanzania	Depression Suicidality	Lack of mental health screening
Nordgreen & Havik, (86)/Arusha, Tanzania	Panic disorder (PD)	Poverty
Nyundo et al., (87)/Dar es Salaam and Dodoma, Tanzania, and other Sub-Saharan Africa	Depression Suicidal ideation Suicidal behaviors	Low socioeconomic status
Patil & Hadley, (88)/Tanzania	Antenatal depression Suicidal ideation	Socioeconomic indicators
Pauley et al., (89)/Kilimanjaro, Tanzania	Depression Alcohol use	Financial stress
Pike & Patil, (90)/Manyara, Tanzania	General mental health Psychological stress Anxiety	Food insecurity/hunger Poverty Lack of animals (cattle) Crop failure Paying taxes No money for basic needs
Prencipe et al., (91)/Iringa and Mbeya,Tanzania	Depression	Lack of economic opportunities Food insecurity Water insecurity
Ramos de Oliveira et al., (92)/Morogoro, Tanzania	Psychosocial development of children	Financial constraints Geographic isolation Lack of trained staff Limited access to mental health services in rural Tanzania
Rogathi et al., (93)/Kilimanjaro, Tanzania	Postpartum depression Non-psychotic mood disorder occurring after childbirth	Antenatal clinics lack mental health screening capabilities Costs of care and transport hinder access Limited availability of psychiatric medications
Rosario et al., (94)/Mwanza, Tanzania	Pregnancy-related anxiety (PRA)	Poor communication in healthcare settings Long wait times at healthcare facilities Financial barriers to care Poverty Inability to afford transportation
Rwakarema et al., (95)/Mwanza, Tanzania	Antenatal depression Pregnancy-related anxiety	Low socioeconomic status No mental health screening in antenatal services Lack of providers trained in mental health care
Rweyemamu et al., (96)/Dodoma, Tanzania	Depression Anxiety Somatic symptoms (headaches, sleep problems, fatigue)	Financial difficulties Job uncertainty Lack of integrated mental health care at the university
Saadi et al., (97)/Dar es Salaam, Tanzania	Post-stroke depressive symptoms	Limited resources for healthcare access Poor access in rural areas Few trained neurologists and mental health professionals
Sajatovic et al., (98) /Dar es Salaam, Tanzania	Chronic psychotic disorders (schizophrenia and schizoaffective disorder)	Inpatient status Poor communication with providers Poverty Long distances to clinics can hinder medication adherence
Saleem et al., (99)/Dar es Salaam, Tanzania	Depression and anxiety among women who use drugs	Low education levels Financial constraints Lack of specialized mental health services
Sariah et al., (100)/Dar es Salaam, Tanzania	Schizophrenia	Financial constraints Cost of atypical antipsychotics Low socioeconomic status Shortage of mental health nurses and specialists
Uriyo et al., (101)/Kilimanjaro, Tanzania	Common mental disorders	Partner with low levels of education

#### 3.3.2 Policy recommendations

**Figure 3:**
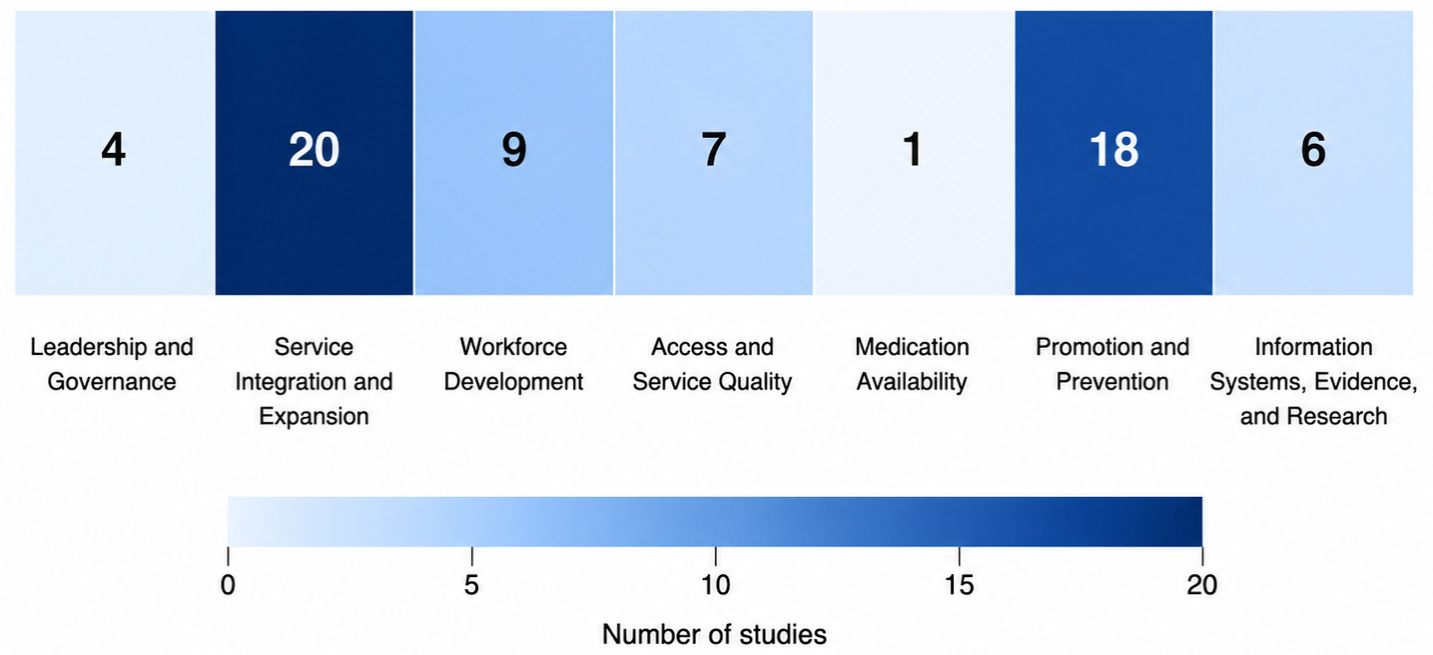
Number of studies with policy recommendations mapped to WHO comprehensive mental health action plan domains.

Among the 44 studies that included policy recommendations, the most frequent recommendations focused on service integration and promotion/prevention, while governance, medication availability, and information systems were much less frequently addressed (Figure 3).

##### 3.3.2.1 Leadership and governance level recommendations

Governance-level recommendations were reported less frequently than recommendations in other domains and focused on strengthening government–provider collaboration to support caregivers and families (47), implementing broader health policies to improve population wellbeing (50), preparing the health sector for disaster-related psychological consequences (71), and tackling stigma while strengthening healthcare infrastructure (95) (Supplementary Table 2).

##### 3.3.2.2 Service integration and expansion

The most widely recommended policies (20 studies) focused on integrating mental health into primary care (31, 72), antenatal and maternal health services (54, 63, 65, 95, 96), community-based and family-oriented care (70, 73, 74), school and university counseling services (56, 69), collaboration with traditional and religious healers (39, 57, 72), and targeted screening in infertility and alcohol-use services (44, 103), and community education and violence prevention and child protection programs in schools (104, 105) (Supplementary Table 2).

##### 3.3.2.3 Workforce development

Recommendations called for integrating mental health training into in-service curricula for doctors, nurses, and primary healthcare workers, and for expanding school-based and university counseling services (14, 57, 63, 64, 75, 95, 96, 106) (Supplementary Table 2).

##### 3.3.2.4 Access and service quality

Engagement of traditional and religious healers as collaborative partners in referral pathways was consistently recommended to improve access and reduce treatment delays (34, 39, 57, 72). Improving geographic access, affordability, outreach, rehabilitation, and follow-up care were also recommended, particularly for perinatal, stroke, and severe mental illness populations (34, 38, 60, 65, 86, 95, 97) (Supplementary Table 2).

##### 3.3.2.5 Medication availability

Only one study addressed the need for a consistent supply of psychotropic medications (48) (Supplementary Table 2).

##### 3.3.2.6 Promotion and prevention

Promotion and prevention recommendations focused on stigma reduction, early screening, and violence prevention. Anti-stigma campaigns targeting communities, health staff, university populations, and police forces were widely recommended (31, 38, 57, 69, 72, 85), alongside early screening for depression in antenatal, postnatal, and chronic disease settings (20, 62, 75, 85) (Supplementary Table 2).

##### 3.3.2.7 Information systems, evidence, and research

Recommendations under information systems, evidence, and research were reported by six studies. They emphasized improving primary healthcare detection of mental disorders (39), adopting culturally sensitive approaches for mental health assessments and interventions (46, 61, 90, 96), and validating tools such as the PHQ-9 for use in Tanzania (97) (Supplementary Table 2).

#### 3.3.3 Relationship between reported determinants and policy recommendations

The relationship between reported determinants and policy recommendation domains is shown in Supplementary Table 3. This comparison showed that health system barriers, such as workforce shortages, screening gaps, weak referral pathways, and limited integration of services, were more often reflected in service-level recommendations. In contrast, structural determinants such as poverty, food insecurity, unemployment, and financial barriers were less consistently connected to recommendations on governance, financing, social protection, or intersectoral policy action.

## 4 Discussion

This scoping review mapped evidence from 74 studies published between 1990, the year of the earliest eligible study identified in the literature search, and June 2025. It addressed two research questions: the structural and health system factors associated with mental health conditions in Tanzania, and the extent to which policy recommendations aligned with the WHO Comprehensive Mental Health Action Plan 2013–2030. In this review, poverty, food insecurity, and financial barriers were the most consistently reported structural determinants of mental health conditions in Tanzania. This finding aligns with broader evidence from sub-Saharan Africa showing that poverty, unemployment, food insecurity, and interpersonal violence are closely associated with poor mental health outcomes (11). However, mental health responses often remain focused on individual-level interventions, leaving the structural conditions linked to poor mental health largely unaddressed. This gap is particularly concerning given that the United Nations Sustainable Development Goals (SDGs) explicitly recognize the interconnection between mental health and broader social determinants: SDG 3 commits to ensuring healthy lives and promoting wellbeing for all, while SDG 1 (no poverty), SDG 2 (zero hunger), and SDG 5 (gender equality) directly target the structural conditions that the included studies most consistently identified as linked to mental ill-health in Tanzania (107). The findings of this review are consistent with this broader pattern at the population level and suggest that policy reform must move beyond clinical service delivery to address the wider social and political conditions that sustain the treatment gap.

### 4.1 Structural and health system barriers

Structural and health system barriers were among the most frequently reported determinants of mental ill-health in the included studies. Among structural determinants, poverty and food insecurity were the most frequently reported factors across the review and appeared across all major condition categories. Because most included studies were cross-sectional, these findings should be interpreted as associations rather than evidence of causality. Poverty, food insecurity, and financial barriers may contribute to poor mental health, but mental health conditions may also worsen economic insecurity by reducing daily functioning, limiting employment, and increasing care-related costs (108, 109). These findings therefore highlight important structural conditions linked to mental health outcomes in Tanzania. Geographic barriers, including long distances to facilities and limited transportation, were also reported in the included Tanzanian studies and disproportionately affected rural and nomadic populations (44, 73). These Tanzania-specific findings are consistent with, but should be distinguished from, broader evidence from sub-Saharan Africa and other LMIC settings. For example, one included Tanzanian study found that severe food insecurity was associated with more than five times higher odds of maternal depression, a pattern that aligns with a systematic review of 64 African studies reporting a dose-responsive relationship between food insecurity and poor mental health (110). Wider SSA literature has also shown that mental health conditions contribute substantially to the regional disease burden, accounting for nearly 20% of total disease burden while receiving less than 1% of national health budgets (111). A review of mental health services across selected SSA countries found that the absence of, or reliance on, outdated mental health policies is associated with poor funding and deteriorating delivery systems, and that Tanzania, despite initiating efforts to incorporate mental health into primary care, has progressed more slowly than comparable countries in the region (3). These external studies provide regional context for interpreting the Tanzanian evidence, but they were not treated as direct findings of this review.

In the Tanzanian context, these barriers have important governance and policy implications. In Tanzania, limited trust in mental health service use and outcomes has contributed to a cycle of underinvestment, poor results, and continued neglect (11). This is reinforced by weak institutional capacity, limited technical support, and fragmented governance between national and district-level authorities. Addressing these barriers therefore requires not only more funding, but also clearer accountability and stronger institutional support for mental health within Tanzania's health system.

The included Tanzanian studies also documented several health system limitations across hospital, primary care, and community settings, including staff shortages, medication stock-outs, inadequate training, lack of routine screening, poor infrastructure, and limited follow-up after discharge (20, 34, 36, 38, 56). Qualitative evidence from Tanzanian health care professionals shows that health workers described missed diagnoses, inadequate training, stigma, and weak referral pathways, including cases where serious mental illness was not recognized or treated in time (11). Lower-level providers often lacked the knowledge, resources, or training needed to identify patients and refer them appropriately. These conditions align with what Patterson terms ‘zones of abandonment', where people with mental illness become functionally invisible within a health system that lacks the capacity and authority to respond (11).

Evidence from other LMIC and SSA settings provides useful comparative support for these Tanzanian findings. A systematic review of barriers to mental health treatment in primary care in LMICs identified shortages of competent primary care professionals with mental health skills, stigma among providers, and the absence of culturally appropriate tools as major barriers to care engagement (112). A study on mental health service integration in Ghana similarly found that stigma among healthcare providers combined with diagnostic deficiencies and inadequate referral systems created compounding structural barriers that persisted despite training efforts (113). These studies are cited here as contextual evidence, rather than as part of the Tanzania-specific dataset.

The absence of validated, culturally adapted screening tools was an additional systemic limitation identified across the included studies. Several included studies relied on instruments originally developed in high-income settings, creating the possibility of systematic measurement bias and underestimating the true prevalence of common mental disorders. This measurement gap is not only a methodological concern but also a structural one: without reliable, locally validated tools, routine screening in primary care cannot function effectively, the evidence base for policy decisions is weakened, and the treatment gap cannot be accurately monitored. The Tanzanian evidence and the wider regional literature suggest that reducing the treatment gap will require coordinated action across financing, workforce development, service delivery, referral systems, medicines supply, and locally appropriate evidence generation.

### 4.2 Policy implications suggested by the included studies

Service integration into primary healthcare and antenatal services was the most widely recommended policy action in this review, followed by promotion and prevention recommendations. The WHO Mental Health Gap Action Program has long identified integration into primary care through task-shifting and task-sharing as the most feasible and cost-effective approach to closing the treatment gap in low-resource settings, where specialist mental health professionals are severely limited (16, 17). A demonstration project using the Mental Health Gap Action Programme intervention guide in Nigeria successfully trained primary care workers and expanded mental health service delivery at the community level, demonstrating that integration is achievable with appropriate governance support (114). A government-led scale-up of task-shifted mental health services in Lagos documented concrete gains across governance, workforce, medicines supply, and service delivery, achieving 64,107 client screenings and reducing medication stock-outs by 42%, providing a practical model for what structured implementation can achieve in a comparable African context (111). Experiences from the programme for improving mental health care (PRIME) and the Africa Focus on intervention research for mental health (AFFIRM) research programs across five LMICs similarly confirmed that integration into primary care is feasible, though its success depends critically on participatory design, stakeholder engagement, and strengthening health-systems alongside clinical training (115). Studies highlight that mental health integration fails not for lack of evidence but because of the absence of governance conditions such as policy support, implementation strategies, workforce capability, and funding arrangements (116), and individual- and organizational-level barriers, such as poor mental health literacy and organizational climate (117). A cross-sectional survey of primary care workers in Ethiopia found that while there was high acceptance of the need to integrate mental health into primary care, workers' willingness to personally deliver such care was substantially lower, highlighting that attitudinal and capacity barriers among providers can undermine even well-designed integration frameworks (118). In Nigeria, contextual factors including chronic underfunding, high staff turnover, and sustained community stigma were found to drive program drift and voltage drop in integrated mental health services, suggesting that adaptive management and governance flexibility are as important as clinical protocol fidelity in low-resource settings (111). Tanzania does not currently have a ring-fenced mental health financing mechanism, and mental health expenditure as a proportion of the national health budget remains negligible (14, 119). Without dedicated financing and governance structures, integration and community-based services will remain fragmented, donor-dependent, and difficult to scale.

This pattern shows a mismatch between the most frequently reported determinants and the types of recommendations proposed. Structural determinants such as poverty, food insecurity, unemployment, and financial barriers were commonly identified, but they were less often matched by recommendations addressing governance, financing, social protection, or intersectoral policy. In contrast, health system barriers such as workforce shortages, screening gaps, referral weaknesses, and service integration challenges were more often translated into recommendations focused on service delivery, training, access, and prevention. This distribution may partly reflect differences in implementation feasibility: service integration can be framed as an actionable response within existing primary care, antenatal, school, university, and community platforms, whereas governance and financing reforms require higher-level institutional authority, political commitment, accountability mechanisms, and sustained resource allocation.

Literature suggests that the role of traditional healers in Tanzania and the wider African context is not straightforwardly negative. A systematic review and meta-analysis by Burns and Tomita (2015) reported that approximately half of individuals seeking formal mental healthcare in Africa choose traditional or religious healers as their first provider and concluded that improving pathways to care must include innovative programs fostering collaboration between biomedical services and community providers (120). A 2024 systematic review of traditional healing practices in SSA found that while there is no conclusive high-level evidence supporting traditional healing alone, studies consistently indicated that collaborative care combining traditional and biomedical services improved mental health outcomes (121). A recent scoping review from traditional healers' perspectives identified five major barriers to integration: differing illness conceptualizations, intellectual property concerns, attitude barriers, weak referral systems, and absence of regulatory frameworks (23). These barriers are directly reflected in the Tanzanian evidence, where policy recommendations called for healer engagement and collaboration but did not propose a regulatory or referral framework to operationalize it. Faith-based mental health providers have also been documented in a systematic review as delivering a range of alternative and complementary care types across 13 African countries, with limited but growing evidence of their effectiveness when integrated into care pathways (122). The evidence therefore supports moving beyond the question of whether traditional healers are part of the Tanzanian mental health system toward the more actionable question of how to structure that collaboration safely, ethically, and effectively.

However, collaboration with traditional healers and faith-based providers should not be understood as straightforward integration into formal mental healthcare systems without safeguards. Important challenges include quality assurance, patient safety, confidentiality, delayed referral, weak referral pathways, and differences in how mental illness is understood and treated (23, 120, 123). Clear roles, basic mental health literacy training, agreed referral mechanisms, supervision, and safeguards against harmful or coercive practices would be essential. In the Tanzanian context, collaboration should therefore be framed as structured and regulated partnership with community providers, while ensuring timely access to evidence-informed, rights-based mental healthcare.

Medication shortages, cost, and limited availability were repeatedly reported as health system barriers across the included studies (36, 38, 100). However, only one study translated this issue into an explicit policy recommendation on ensuring a consistent supply of psychotropic medicines (48). This discrepancy likely reflects the difference between documenting a health system problem and formulating a specific policy response. Because this review coded only author-reported recommendations, medication barriers were not recoded as recommendations unless the original authors explicitly proposed action on medicine supply.

This evidence-to-recommendation gap may have several explanations. First, some studies may have documented medication shortages as background service barriers without examining procurement, supply-chain management, or pharmaceutical financing in sufficient depth to support specific policy recommendations. Second, researchers may have viewed medicine supply as a higher-level health system or government procurement issue outside the immediate scope of clinically oriented or population-based mental health studies. Third, the wider orientation of much mental health research toward screening, prevalence, service use, and clinical integration may make service-delivery recommendations more visible than recommendations concerning financing, procurement, and accountability. These interpretations should be treated cautiously, as this review did not formally assess authors' motivations, funding arrangements, or political context. Nevertheless, the finding suggests that psychotropic medication access has been recognized as a barrier in the Tanzanian literature but has been less consistently developed into concrete policy recommendations.

This gap is important because reliable access to essential psychotropic medicines is necessary for effective service integration. A scoping review protocol of integrated primary mental healthcare in LMICs similarly identified poor medicine availability as one of the main barriers to optimal integration, alongside workforce shortages and high workloads (124). Without a dependable medicines supply, trained providers cannot deliver effective pharmacological care even when patients are identified and referred.

Research and information systems, corresponding to WHO MHAP Objective 4, were also underrepresented, with only six studies recommending validation of locally appropriate screening tools. Gronholm et al. have argued for multilevel strategies that include strengthening information systems as a prerequisite for stigma reduction and service improvement in LMICs (125). Stowell et al. identified validation of culturally adapted assessment tools, longitudinal research designs, and intersectoral research partnerships as key priorities for the mental health research agenda in SSA (126).

From a socio-ecological perspective, the distribution of policy recommendations in this review concentrates mainly at the middle levels of the framework, particularly service delivery and community promotion, while leaving the structural foundations of governance, financing, and research infrastructure less developed. Tanzania's mental health progress will depend not only on what is done in clinics and communities, but also on whether the political, institutional, and research conditions needed to sustain those efforts are built and maintained alongside clinical investment.

### 4.3 Strengths and limitations

This review has several limitations that should be kept in mind when interpreting the findings. Most included studies were cross-sectional, which limits causal inference and prevents definitive conclusions about the direction of associations between structural factors and mental health outcomes. Many also used screening tools not originally developed for Tanzanian populations, raising the possibility of measurement error or under-identification of some conditions. Self-reported data on sensitive topics such as intimate partner violence and suicidal ideation may also have been affected by underreporting due to stigma and fear of judgment. The studies were heavily concentrated in a few urban and semi-urban regions, particularly Dar es Salaam, Kilimanjaro, and Mwanza. This means that people living in more remote or rural parts of Tanzania are not well represented in the evidence. This geographic concentration may reflect the location of research infrastructure and referral services, but it may also reflect publication and indexing bias, with studies from academic centers and internationally indexed journals being more visible than locally produced evidence. The review included only English-language peer-reviewed journal articles indexed in the selected databases. As a result, relevant studies published in Swahili, government reports, NGO evaluations, dissertations, and other gray literature may have been missed. African regional databases were also not searched because they were not accessible to the review team. We were unable to quantify how much Tanzanian mental health evidence may have been missed, because there is no comprehensive denominator for unpublished, non-English, locally indexed, or gray literature sources. However, this limitation may have skewed the findings toward urban, academic-center, and internationally published research, while underrepresenting rural, community-based, service-delivery, and implementation evidence. Future reviews could strengthen and enrich this evidence base by including Swahili-language sources, African regional databases, government and NGO reports, and locally produced scholarship.

Despite these limitations, this review has several strengths. To our knowledge, this is the first scoping review to map Tanzania-specific mental health evidence on structural determinants and policy recommendations against the WHO Comprehensive Mental Health Action Plan. The search was conducted systematically across five major databases with support from specialist librarians, and studies were screened independently by multiple reviewers, which helped reduce selection bias. The involvement of Tanzanian researchers throughout the review also strengthened the interpretation of findings by grounding the analysis in local context, culture, and health system realities.

## 5 Conclusion

This scoping review provides the most comprehensive mapping to date of published evidence on mental health conditions, structural and health system determinants, and policy recommendations in Tanzania. The findings suggest that poverty, food insecurity, financial barriers, and health-system weaknesses interact in ways that sustain the mental health treatment gap. Addressing this gap requires responses that are culturally responsive, structurally informed, and integrated across community and health system settings.

The recommendations identified in the included studies mainly emphasized integration of mental health into primary and antenatal care, engagement of traditional and religious healers in referral pathways, and community-level promotion and prevention. In contrast, governance reform, sustainable mental health financing, reliable psychotropic medicine supply, and culturally adapted and validated screening tools were addressed less often. This pattern suggests that the literature has focused more on service- and community-level responses than on broader system-level solutions.

Future research should prioritize longitudinal and intervention studies, validation of locally appropriate assessment tools, and implementation-focused research that can better inform national policy and service development. However, as the WHO Comprehensive Mental Health Action Plan approaches its 2030 endpoint, Tanzania also needs to move from evidence generation toward implementation of system-level reforms. Based on the recommendations reported in the included studies, the Ministry of Health is well-positioned to lead service integration, workforce planning, medicines availability, and mental health information systems, while PO-RALG and local authorities support district and community implementation. The Ministry of Finance and other relevant sectors, including education, social welfare, agriculture, and gender-related policy, should contribute to sustainable financing and action on structural determinants. International donors are encouraged, based on the patterns identified in this review, to align support with national priorities by investing in governance capacity, routine data systems, workforce development, and reliable essential medicines supply.

## Data Availability

The original contributions presented in the study are included in the article/supplementary material, further inquiries can be directed to the corresponding authors.
