# Supplementary material for: Structural and health system determinants of mental health in Tanzania: mapping policy recommendations to the WHO comprehensive mental health action plan 2013–2030
Source: Front Public Health. 2026 Jul 10;14:1878653. doi: 10.3389/fpubh.2026.1878653 (PMC13398205; doi:10.3389/fpubh.2026.1878653)
Supplement: Supplementary file 1 [file Supplementary_file_1.docx]

**Supplementary Table 1. Characteristics of the Included Studies (n = 74).**

| **Author / Year / Region** | **Study Design / Context** | **Sample** | **Data Collection** | **Key Findings** | **Limitations** |
| --- | --- | --- | --- | --- | --- |
| Adams et al., 2021  Kilimanjaro, Tanzania [31] | Cross-sectional study  Community | n = 304  Elders aged ≥60 years | Face-to-face interviews using a structured questionnaire | Elders with self-reported cognitive impairment had higher prevalence of depressive symptoms (PR = 1.66, 95% CI 1.16–2.38). Those with intermediate (PR = 0.56, 95% CI 0.38–0.82) and strong social support (PR = 0.27, 95% CI 0.17–0.44) were less likely to have depressive symptoms compared to those with no available social support. | No validated tool (GDS-15 not validated in Tanzania).  Self-reported cognitive impairment; possible misclassification bias. |
| Alemu et al., 2020  Zanzibar, Tanzania [32] | Prospective matched cohort study  Both hospital and community | n = 488  Postpartum women | Structured interviewer-administered questionnaire:  PHQ-9 (depressive symptoms)  HTQ-16 (PTSD symptoms)  WHOQOL-BREF (quality of life, 4 domains)  HSCL-25 (social support domain) | PHQ-9: Median = 1 (IQR 0–3); ~7% mild depression, none moderate/severe. HTQ-16: All below cut-off of 2.5 (no PTSD cases). WHOQOL-BREF: High scores across domains — Physical (85.7–96.4), Psychological (95.8–100), Social (75–100), Environmental (87.5–93.8). Trajectories: Depression stable across groups; PTSD symptoms improved in controls but remained stable in MNM women; physical QoL started lower in MNM but improved faster over time. | Sample size may not detect small associations.  Dropouts could introduce unmeasured bias.  Tools not validated in the Zanzibar context.  Outcomes only measured post-discharge.  Possible unmeasured confounders (e.g., prior pregnancy loss, nutrition). |
| Aloyce et al., 2024  Mwanza, Tanzania [33] | Cross-sectional study  Community | n = 828  Young men aged 18–24 years, residing in Mwanza ≥3 months | Instruments/Measures:  Depression: PHQ-9 (Swahili validated)  Suicidal thoughts: PHQ-9 item + 2 CoVAC items | Depression significantly associated with sexual IPV (Adjusted OR 2.19, 95% CI 1.57–3.04), emotional IPV (AOR 1.76, 95% CI 1.26–2.46), controlling behaviors (AOR 1.46, 95% CI 1.10–1.93), and economic IPV (AOR 1.32, 95% CI 1.01–1.74). After adjustment, no IPV type remained significant.  Other risk factors: Food insecurity (AOR 3.00, 95% CI 2.19–4.10); harmful alcohol use (AOR 2.19); dependent alcohol use (AOR 3.43); tobacco/substance use (AOR 2.00); gambling (AOR 1.75 for depression; AOR 2.44 for suicidal thoughts). | Cross-sectional design (no temporality).  Non-clinical mental health measures.  Generalizability limited to Mwanza.  Crude measurement of suicidal thoughts.  Variation in measurement of economic partner violence.  Adverse childhood experiences unanalyzable (nearly universal). |
| Ambikile & Iseselo, 2017 Dar es Salaam, Tanzania [36] | Cross-sectional qualitative study  Hospital | n = 30  Patients with chronic mental illness  Caregivers of mentally ill patients  7 mental health care providers  7 patients with chronic mental illness  16 caregivers | In-depth interviews and focus groups conducted in Kiswahili; audio-recorded with semi-structured guides | Resource challenges: Inadequate training, staff shortages, lack of wards, poor infrastructure, limited equipment, drug shortages.  System challenges: Misallocation of trained staff, harsh language, long waits, short consultations, rising patient load, weak managerial support. | Limited scope of participants.  Cross-sectional design — one-time qualitative snapshot; cannot capture changes or trends over time.  Convenience sampling — participants recruited based on availability during clinic days; possible selection bias and limited representativeness.  Small sample size — only 7 providers, 7 patients, and 16 caregivers; restricts generalizability.  Self-reported data — interviews and FGDs may be affected by recall or social desirability bias. |
| Ambikile et al., 2023  Dar es Salaam, Tanzania [34] | Descriptive qualitative study  Hospital | n = 29  7 patients with chronic/severe mental illness  16 caregivers (8 female, 8 male)  6 healthcare providers (3 nurses, 2 assistant medical officers, 1 district mental health coordinator) | In-depth interviews (IDIs) with patients and healthcare providers.  Focus group discussions (FGDs) with caregivers (2 groups, 8 participants each). | Limited knowledge, stigma, cultural beliefs, family neglect, and weak outreach systems create major barriers to effective home care for people with severe mental illness. | Small purposive sample (29 participants); limits generalizability.  Included only patients, caregivers, and providers; excluded hospital managers and community members.  Cross-sectional design — captures experiences at one point in time.  Sensitive issues may not have been fully disclosed in focus groups. |
| Ambikile et al., 2012  Dar es Salaam, Tanzania [35] | Cross-sectional study  Hospital | n = 19  Children ≤12 years (with autism, ADHD, seizure disorders, epilepsy, learning disabilities, mental retardation)  Caregivers included mothers, fathers, and one grandmother | Focus groups; semi-structured in-depth interviews | Caregivers faced high psychological distress, social stigma and exclusion, and heavy economic strain while caring for children with mental disorders. | Results are context-specific, not generalizable, limited to caregiver perspectives, and shaped by qualitative sampling. |
| Bintabara et al., 2024  Dar es Salaam, Dodoma, Mbeya, Morogoro and Mwanza, Tanzania [37] | Cross-sectional study  Urban, higher-education settings | n = 25  Medical students | Online self-administered questionnaire using the Depression, Anxiety and Stress Scale-21 (DASS-21) | Depression (28.9%), anxiety (54.1%), and stress (15.1%) clinically significant cases; any mental disorder combined (58.6%).  Predictors (AOR): 4th year (AOR=5.99) and 5th year (AOR=5.52) → higher depression risk. Married/cohabiting → higher depression (AOR=1.84) and anxiety (AOR=2.35). Private university students → higher anxiety (AOR=2.32) and stress (AOR=2.90). Clinical rotation → higher odds of any mental disorder (AOR≈1.6–1.7). | Cross-sectional design limits causal inference.  DASS-21 may not fully capture cultural nuances in Tanzania.  Dichotomization may misclassify prevalence.  COVID-19 medical student sample limits broader applicability. |
| Blixen, 2020  Dar es Salaam, Tanzania [38] | Cross-sectional study  Hospital | n = 44  15 patients with chronic psychotic disorders (schizophrenia or schizoaffective disorder)  14 family caregivers (mostly mothers, fathers, siblings; majority women)  15 healthcare providers (psychiatrists, psychiatric nurses, occupational therapists, clinical psychologist) | Semi-structured interviews (English→Swahili), audio-recorded; member-checking | Patients, family caregivers, and healthcare providers described many barriers to managing chronic psychotic disorders, including limited knowledge, cultural beliefs, psychological stress, harmful behaviors, and weak health system support.  Stigma, stress, and depression worsened the illness experience and made it harder to take medication consistently.  Religious and traditional beliefs strongly shaped help-seeking and sometimes led to stopping hospital treatment.  Long-acting injectable antipsychotics were mostly viewed positively for convenience, better adherence, and reducing stigma, though concerns remained about side-effects, cost, and availability. | Single-city convenience sample.  No back-translation.  Possible self-report bias.  Transferability limited. |
| Bondestam et al., 1990  Zanzibar, Tanzania [39] | Cross-sectional study  Community | n = 10,776 individuals  General population (5,423 males and 5,353 females) | Structured household interviews with symptom checklists, followed by clinical validation | 59 people with mental disorders detected (5.5/1,000). Epilepsy prevalence: 4.9/1,000 (highest in children 0–14 years). Chronic psychosis: 2.6/1,000 (schizophrenia 0.6/1,000). Acute psychosis: 0.6/1,000.  Treatment use: About 1 in 8 patients used only biomedical care; majority combined traditional and modern treatment; one-third used only traditional healing.  Hospital admissions: 16 patients (mostly young/middle-aged men with higher education). | Underreporting.  Cultural bias.  Missing mild cases.  Reliance on symptom descriptions.  Cross-sectional design limits inference. |
| Cherewick et al., 2024  Dar es Salaam, Tanzania [40] | Cross-sectional study  Community | n = 350  Very young adolescents aged 10–15 years, orphans | Survey interviews (75–90 minutes):  Childhood Trauma Scale  KidCope Questionnaire (adapted with added prayer item)  African Youth Psychological Assessment (AYPA)  Everyday Discrimination  Pubertal Development Scale (PDS) | Resignation coping — higher depression, anxiety, externalizing. Emotional neglect — higher depression and anxiety. Emotional abuse — higher depression. Stigma/discrimination — higher depression, anxiety, externalizing. Female sex — higher anxiety (small effect). More advanced puberty — higher depression (baseline only). | Cross-sectional design — cannot show causal relationships or long-term effects of coping strategies.  Findings are context-specific to Tanzanian adolescent orphans.  Coping responses may vary depending on stressor type or social relationships.  Self-reporting may have introduced response or social desirability bias. |
| Daniel et al., 2018  Arusha, Tanzania [41] | Exploratory qualitative study  Community | n = 41  Modern healthcare providers, religious leaders, traditional practitioners, local government leaders, local Maasai leaders, and workers from NGOs dealing with mental health (21 females, 20 males) | In-depth interviews; focus group discussions; semi-structured interviews; recording and note-taking | Participants attributed mental disorders to supernatural causes (curses, witchcraft, demons, God's will). A few mentioned biological causes and risk behaviors (perinatal insults, head injuries, drug abuse). The Maasai community seeks mental health care in a sequential and simultaneous manner from professional health-care providers, traditional healers, and religious leaders. Traditional healers and religious leaders were preferred over professional health-care providers. | Cross-sectional design limits understanding of changes over time.  Findings specific to the rural Maasai community in Monduli District.  Translation from Maasai to Kiswahili may have resulted in loss of nuance.  The sensitive topic of mental health and associated stigma may have restricted open discussion, particularly in focus groups.  Qualitative study — findings are context-specific and not statistically generalizable. |
| Decaro et al., 2016  Mwanza, Tanzania [42] | Cross-sectional study  Community (regional health centers) | n = 88  Mother–infant dyads | Surveys and interviews:  Household Food Insecurity Access Scale (HFIAS)  MacArthur Subjective Social Status Scale  Hopkins Symptom Checklist (HSCL-25)  Household wealth inventory | Severe food insecurity (OR: 5.16), lower subjective social status (r = −0.32), and lower household wealth (r = −0.26) were associated with high symptoms of maternal depression. Lower household wealth (r = −0.21) and severe food insecurity (OR: 2.52) were associated with high anxiety. | Cross-sectional design limits causal inferences between variables.  Small sample size reduces generalizability.  Self-reported measures are potential source of bias in maternal mental health data. |
| Dorsey et al., 2015  Kilimanjaro, Tanzania [43] | Exploratory and descriptive qualitative study  Community | n = 107  Orphaned children (7–13 years), their guardians (mainly women), and key informants (community leaders and teachers)  Free Listing Interviews: 73 participants (36 guardians and 37 orphaned children aged 7–13)  Key Informant interviews (3 key mental health issues explored) | Free Listing (FL) Interviews: Participants listed problems faced by orphans, focusing on mental health-related issues.  Key Informant (KI) Interviews: Explored three key mental health issues (mistreatment/abuse, not feeling loved, stress/overthinking). | Mistreatment/abuse linked to loneliness, sadness, and behavioral issues. Not feeling loved is associated with low self-esteem and hopelessness. Stress/overthinking caused by deprivation, leading to emotional distress and cognitive challenges. | Handwritten notes: Interviews were not recorded, increasing the risk of inaccuracy.  Key Informant interviews addressed only three mental health problems, limiting the scope of findings. |
| Herlosky et al., 2020  Mara, Tanzania [44] | Cross-sectional study  Community | n = 23  Mothers with infants less than 1 year (age 15–40 years) | Psychometric-tested instrument; semi-structured interviews | No systemic differences based on residency, whether women resided in bush camps or in camps located closer to villages. No correlation between levels of social support and increased depressive scores. | Methods of translation of the EPDS.  Limited sample size. |
| Hill et al., 2020  Dar es Salaam, Tanzania [45] | Cross-sectional study  Community | n = 1,249  Young men in social groups | Interviews using questionnaires; HSCL-25; Filmer PW Index (socioeconomic status) | Anxiety and depression were related to living apart from family, employment, and childhood experience of violence. | Limitation to the instruments (developed in other countries and settings) and the cross-sectional design. |
| Hovland et al., 2025  Tanga, Tanzania [20] | Qualitative descriptive study  Community | n = 12  Caregivers (3 men and 9 women) | Semi-structured in-depth interviews | Caregivers experienced violence, lack of understanding of the needs of the mentally ill, lack of support, and challenges in access to resources. Caregivers sought help from religious prayers, traditional healers, professional facilities, and biomedical medication. | Limited to the district of Tanga. |
| Howorth et al., 2019  Kilimanjaro, Tanzania [46] | Qualitative study  Community | n = 81  Older people over 60 years (37 male and 44 female over 60 years of age) | Focus groups (n=10) | Main themes: conceptualization of depression by older people and differentiation from other related conditions ("too many thoughts," cognitive, affective and biological symptoms, wish to die, somatic symptoms); the causes of depression (inability to work, loss of physical strength and independence, lack of resources, family difficulties, chronic disease); management of depression (love and comfort, advice, spiritual support, medical help). | Study design, participants able to attend, interview method used influenced the discussions.  The interviewers themselves all had the potential to influence the discussions; and the same limitation is suggested for the framing of the inquiry, questions asked, and the use of vignettes presented.  Case vignettes based on cases seen locally by members of the team with a clinical psychiatry background aimed to reduce any inaccuracies. |
| Iseselo et al., 2020  Dar es Salaam, Tanzania [49] | Qualitative, descriptive study  Community | n = 35  6 patients with mental health diagnosis  16 caregivers  13 community members | 4 focus groups (2 with caregivers, 2 with community members);  6 in-depth interviews with patients | Four themes: promoting patients' participation in household activities; improving patients' support system; patients' involvement in home activities; promoting self-care management; improving patients' support systems; providing safety and protection are important factors that promote recovery for people with mental illness. | Not carrying out in-depth interviews with caregivers and non-involvement of mental health care providers. This could elicit more information about patients' needs and their experiences than it was captured in patients. |
| Iseselo et al., 2017  Dar es Salaam, Tanzania [48] | Qualitative, descriptive study  Hospital | n = 27  Patients with mental health diagnosis (2 male, 2 female)  Caregivers (7 male, 9 female)  Mental health care workers/nurses and social workers (5 male, 2 female: 25–55+ of age) | Focus groups and in-depth interviews | Promoting patients' self-care management and providing safety and protection among patients with mental illness. | Just one district in Tanzania.  Lack of psychiatrists in the sample.  Lack of pharmacists to evaluate medication. |
| Iseselo et al., 2016  Dar es Salaam, Tanzania [47] | Explorative qualitative study  Hospital | n = 14  Caregivers (5 male, 9 female) [age 35–60 years] | Focus groups (4) and in-depth interviews (2) | Financial constraints, lack of social support, disruption of family functioning, stigma, discrimination, and disruptive behavior. Families and the patients are left alone without any kind of follow-up. | Just one city in Tanzania; however, the largest.  Small number of participants.  Only one primary caregiver (who spent most of the time with the patient) was recruited.  Hospital-based — findings are not generalizable to a community-based sample. |
| Ivanova et al., 2022  Mbeya and Songwe, Tanzania [50] | Cross-sectional study  Community and hospital | n = 393  Adults (>18 years) with mental health diagnoses | Face-to-face interviews with questionnaires assessing health-related quality of life (SF-36) and K10 for measuring psychological distress | Older participants (≥40 years) and those who were divorced/widowed reported lower physical functioning, energy/vitality, and emotional well-being compared to their counterparts (p<0.05). Majority of participants (78.4%; 305/389) reported likely to be well (K10 score<20), while 13.4% (52/389) reported mild (K10 score 20–24), 5.7% (22/389) moderate (K10 score 25–29), and 2.6% (10/389) severe (K10 score≥30) psychological distress. | Limited to five districts in Tanzania.  Self-reported.  Health status assessed subjectively using a questionnaire with special focus on absence of respiratory or cardiac diseases.  Questionnaire primarily designed in English and verbally translated to participants by trained staff, which could lead to some bias related to understanding of the questions. |
| Jenkins et al., 2010, Dar es Salaam, Tanzania [52] | Population-based study  Community | n = 899  Adults (age 15–59 years) | Psychotic Screening Questionnaire; Clinical Interview Schedule Revised; Alcohol Use Disorder Identification Test; sociodemographic self-rating | 35 (3.9%) respondents endorsed one or more PSQ items. The annual prevalence of psychotic symptoms was significantly lower in the middle income Ilala compared to the more densely populated Saba Saba (2.1 vs. 6.0%, unadjusted OR = 0.33; 95% CI 0.16–0.70, p = 0.004). "Strange experiences" were the most reported symptoms in both areas. | Lack of supervision on the logistics of the project.  Instruments not designed for sub-Saharan Africa.  Low sample size.  Psychosis diagnosis was not confirmed by trained psychiatrists. |
| Jenkins et al., 2010  Dar es Salaam, Tanzania [51] | Cross-sectional study  Community | n = 899  Adults of Ilala-Shaurimoyo and Mtoni-Saba Saba (aged 15–59) | Clinical Interview Schedule Revised (CIS-R) for CMD; Psychosis Screening Questionnaire (PSQ); Alcohol Use Disorders Identification Test (AUDIT); demographic and socioeconomic questionnaire; social support and social network questions | CMD is 39.8 times higher among adults with ≥3 stressful events; 8.9 times higher in adults with relationship problems; 6.63 times higher among income-unstable adults; and 2.31 times higher among adults with death of a loved one in the 6 months. There was no significant gender difference in prevalence of CMD. | Findings limited to two areas in Dar es Salaam.  Some missing data from paper CIS-R.  Possible underestimation of prevalence.  In the regression analyses, selection probabilities are not weighted. |
| Jeong et al., 2024  Mwanza, Tanzania [53] | Qualitative study  Community | n = 120  56 mothers and 56 fathers of children under 2 years; 8 community leaders; community health workers | In-depth interviews and group discussions | Mental health symptoms, causes, negative coping strategies (alcohol, violence, withdrawal), and positive coping strategies (partner support, peer/family support, exercise, religion). This study highlights the importance of supporting positive mental health among fathers. | Generalizability limited to Mwanza and co-residing fathers with their partners and children; non-residential/single fathers excluded.  Limited in-depth analyses of specific themes pertaining to paternal mental health. |
| Kaaya et al., 2010  Dar es Salaam, Tanzania [54] | Mixed methods study — formative qualitative, unstructured interviews, and a prospective survey | n = 600  Pregnant women in second and third trimester | Unstructured interviews; Kiswahili adapted version of the Hopkins Symptom Checklist (KHSCL); Rost two-item screener | Ever experiencing a depressive episode was the strongest risk factor, increasing almost four-and-a-half-fold the likelihood of reporting depressive symptoms (OR = 4.35; 95% CI: 2.66, 7.11). Low and moderate satisfaction with ability to access basic needs both increased the likelihood of depressive symptoms about two-fold (OR = 2.18; 95% CI: 1.38, 3.27 and OR = 1.86; 95% CI: 1.01, 3.34, respectively). Early booking for antenatal care or misunderstandings/conflicts with respondent's partner in the year prior to assessment each increased by almost two-fold the likelihood of reporting depressive symptoms (OR = 1.87; 95% CI: 1.09, 3.22 and OR = 1.89; 95% CI: 1.21, 2.95, respectively). | Cross-sectional at baseline — limits inference on onset/timing.  Possible overestimation of depression measured by screening tool.  Recall bias possible for psychosocial risk factors. |
| Khan et al., 2019  Dar es Salaam, Tanzania [55] | Descriptive cross-sectional study  Hospital | n = 3,013  Out-of-school adolescent girls and young women (15–23 years old) | Audio Computer-Assisted Self-Interview (ACASI) using structured questionnaire; Patient Health Questionnaire (PHQ-4), composed of PHQ-2 (depression screener) and GAD-2 | Being a current smoker and being on insulin therapy were independent predictors of mild to moderate depression among diabetic patients. Patients on insulin therapy were almost twice as likely to have mild to moderate depression (OR 1.78 [95% CI 1.12–2.82], p = 0.015). Current smokers were almost seven times more likely to have mild to moderate depression (OR 6.72 [95% CI 1.26–35.70], p = 0.025). | Hospital-based, single-centre study — may not generalize to all Tanzania.  Exclusion of patients currently on depression treatment.  PHQ-9 is a screening tool, not diagnostic. |
| Knettel, 2018  Arusha and Kilimanjaro, Tanzania [56] | Qualitative study  Psychiatric inpatient and outpatient clinic; School special education; Psychiatric rehabilitation village | n = 29  Mental health providers (nurses, psychotherapists, psychiatrists, special educators, occupational therapist, drug counselor) | Open-ended, semi-structured interviews | Understanding of mental health treatment in Tanzania through providers' perspectives on diagnostic and treatment framework. | Small sample size.  Limited to two regions in Tanzania.  Not generalizable.  Study included only provider perspectives, not patients/community. |
| Knettel et al., 2018  Arusha and Kilimanjaro, Tanzania [57] | Qualitative study  Psychiatric inpatient and outpatient clinic; School special education; Psychiatric rehabilitation village | n = 29  Mental health providers (nurses, psychotherapists, psychiatrists, special educators, occupational therapists, drug counselor) | Semi-structured in-depth interviews | Interviewees acknowledged critical shortfalls in providers, facilities, or resources dedicated to mental health; little coordination among services; and multiple barriers that prevent all but the most severely impaired patients from receiving care. Despite these challenges, the majority of providers believed that treatment would improve in the future. | Self-report bias (providers may have given socially desirable answers).  Limited to two regions and formal providers only.  Results not generalizable to all Tanzania.  Small sample size. |
| Kuringe et al., 2019  Shinyanga, Tanzania [58] | Cross-sectional study  Community | n = 787  Antenatal women between ages 15–48 (gestational age 28–32 weeks) | Kiswahili-translated HSCL-25; Kiswahili version of SF-36 | Using the PHQ-4 tool, 33% (95% CI 30.8%–34.2%) had mild, 20% (95% CI 18.3%–21.1%) moderate, and 6% (95% CI 5.5%–7.2%) severe symptoms of anxiety and depression. Violence experience from sexual partners (AOR = 1.63, 95% CI: 1.36–1.96) and HIV-positive status (AOR = 1.54, 95% CI: 1.03–2.31) were strongly associated with anxiety symptoms. Living alone with younger siblings or others (AOR = 2.51, 95% CI: 1.47–4.29) and violence from sexual partners (AOR = 1.90, 95% CI: 1.59–2.27) were strongly associated with depression symptoms. Having savings (AOR = 0.81) and emotional support (AOR = 0.82) were protective. | Cross-sectional design (no causality).  Use of not-validated tool (PHQ-4 not validated in Tanzania).  Self-reported data bias.  No data on household clustering or duration of out-of-school status.  Absence of qualitative data. |
| Lee et al., 2008  Dar es Salaam, Tanzania [59] | Validation study  Primary care | n = 1,047  Undergraduate students | Self-administered questionnaire; PHQ-9 for depression (all students); SRQ-20 (second cohort); SCOFF for eating disorders (second cohort) | HSCL-25 showed good internal consistency (α = 0.90 total; depression α = 0.88; anxiety α = 0.76); test-retest reliability = 0.85; correlations with SF-36 dimensions (r = −0.35 to −0.55) confirmed discriminant validity. | Findings not generalizable.  No gold standard clinical validation.  Lack of calibration for local cut-offs.  Self-report limitations.  Absence of ethnographic validation. |
| Lugata et al., 2021  Kilimanjaro, Tanzania [60] | Cross-sectional study  University | n = 1,007  Pregnant women receiving antenatal care | WHO IPV tool (emotional, physical, sexual violence); Connor-Davidson Resilience Scale (CD-RISC-10); Edinburgh Postpartum Depression Scale (EPDS) | Being in year 2 carried an Odds Ratio for depression of 1.9 (AOR = 1.9, 95% CI =1.2–3.0), rising to 2.1 (AOR = 2.1, 95% CI = 1.3–3.6) for those in year 3 when compared to students in year 1. Students reporting regular use of alcohol, tobacco, or hard drugs (107 students in total) had an increased risk of depression (AOR = 1.8, 95% CI = 1.1–3.1). Those reporting an unhappy relationship with a partner had an AOR for depression of 1.8 (95% CI = 1.2–2.6). Students declaring chronic physical illness (4.2%) also had significantly increased risks of depression (AOR = 2.8, 95% CI = 1.3–6.1), while those considering dropping out had an increased odds for depression (AOR = 1.5, 95% CI = 1.1–2.2). Not having sufficient time to socialize with friends was associated with depression (AOR = 1.7, 95% CI = 1.1). | Cross-sectional design (no causality).  Timing of surveys strongly influenced prevalence.  Reliance on self-reports. |
| Magnusson et al., 2021  Kilimanjaro, Tanzania [61] | Cross-sectional study  Antenatal care clinics | n = 3,013  Out-of-school adolescent girls and young women (15–23 years old) | Audio Computer-Assisted Self-Interview (ACASI) using structured questionnaire; PHQ-4 (comprising PHQ-2 and GAD-2); CD-RISC-10; EPDS | IPV strongly associated with prenatal depression: aOR any intimate partner violence = 6.49 (95% CI 3.75–11.24); physical intimate partner violence aOR = 13.20 (95% CI 6.11–28.53). Resilience mean score = 14.26 (SD 9.45), not associated with prenatal depression. | Cross-sectional design (causality issues).  No validation of CD-RISC-10 Swahili version.  Reliance on self-reported IPV and depression (risk of recall/social desirability bias).  EPDS cut-off issues.  Low resilience scores raise validity concerns. |
| Mahenge et al., 2013  Dar es Salaam, Tanzania [63] | Cross-sectional study  Hospital | n = 1,180  Pregnant women attending antenatal care aged 17–43 years | Questionnaire; The Conflict Tactics Scale; the John Hopkins Symptom Checklist (25); Posttraumatic Diagnostic Scale | Women who experienced physical and/or sexual intimate partner violence during pregnancy were significantly more likely to have moderate PTSD (AOR 2.94, 95% CI 1.71–5.06), anxiety (AOR 3.98, 95% CI 2.85–5.57), and depressive (AOR 3.31, 95% CI 2.39–4.593) symptoms. | Cross-sectional design (no causal inference).  Potential underreporting of experiences of intimate partner violence and mental health symptoms.  Bias of clinic-based sample (urban — who have access to antenatal care).  Not diagnostic tools used.  PDS not validated in Tanzania. |
| Mahenge et al., 2015  Dar es Salaam, Tanzania [64] | Cross-sectional study  Hospital | n = 1,180  Pregnant women attending prenatal care (aged 17–43 years) | Questionnaire; the Hopkins Symptoms Checklist and the PTSD Diagnostic Scale | Self-employment increased anxiety (AOR=1.56) and depression (AOR=1.52); older women (35–43 years) had lower anxiety (AOR=0.60); college education protective for depression (AOR=0.54); not married/cohabiting predicted higher PTSD (AOR=2.71); short relationship duration reduced anxiety and PTSD risk. | Cross-sectional (no causality).  Non-diagnostic screening tools.  PDS not validated in Tanzania.  Urban tertiary setting limits generalizability.  Stigma and social desirability bias. |
| Mahenge et al., 2018  Dar es Salaam, Tanzania [62] | Cross-sectional survey  Postnatal care clinics | n = 500  Postpartum women (aged 18–48 years) attending postnatal clinics 1–9 months after delivery | Questionnaire; Adverse Childhood Experiences (ACE) scale; WHO IPV scale; PHQ-9 (postpartum depression) | Symptoms of postpartum depression were reported by 13.6% (68) of women at one month to nine months postpartum. Physical ACE: 32.8%; Sexual ACE: 11.6%; Psychological ACE: 25.6%; Any ACE: 49.2%; Physical/sexual IPV during pregnancy: 18.8%. | Done in health centers in Dar es Salaam — results cannot be generalized to the rest of Tanzania's population.  Reporting bias due to the sensitive nature of the study (underreporting of violence or depression symptoms due to embarrassment or stigma).  Issues with recall bias.  Study did not capture other forms of ACEs and IPV which might affect the mental health of the women as well. |
| Manongi et al., 2020  Kiliamnjaro, Tanzania [65] | Cross-sectional study  Antenatal care | n = 1,116  Pregnant women | Edinburgh Postpartum Depression Scale (EPDS) | 433 (38.8%) were exposed to at least one type of violence during their pregnancy, and 128 (11.5%) presented with signs of depression. The most common type of violence experienced was emotional violence (30.7%), followed by sexual violence (19.0%) and physical violence (10.0%). Exposure to at least one type of violence was the strongest predictor for depression (AOR = 5.06; 95% CI = [3.25, 7.86]), followed by women who reported their primary source of emotional support was individuals not related to their family (AOR = 2.25; 95% CI = [1.26, 4.02]). Positive HIV/AIDS status (AOR = 2.27; 95% CI = [1.01, 5.14]) and previous history of depression (AOR = 1.62; 95% CI = [1.00, 2.64]). Physical violence was the strongest predictor for signs of depression after adjusting for types of violence (AOR = 4.42; 95% CI = [2.65, 7.37]). | EPDS used to measure signs of depression not validated by a psychiatrist to set an EPDS cutoff score which confirms depression.  Some women may underreport the occurrence of violence.  The cross-sectional study design could lead to difficulty in interpreting the direction of causality.  Large cultural differences between communities across Tanzania — findings can be generalized only to communities outside Kilimanjaro Region. |
| Massae et al., 2021  Pwani, Tanzania [67] | Cross-sectional survey  Health facilities | n = 694  Pregnant women | Wijma Delivery and Expectation Questionnaire version A (W-DEQ-A) and Edinburgh Postnatal Depression Scale (EPDS) | The overall prevalence of Fear of Birth (FoB) was 15.1% and that of Depressive Symptoms (DS) was 17.7%. About 37 (5.3%) of pregnant women had both FoB and DS, 68 (9.8%) had FoB but no DS, 86 (12.4%) had DS but no FoB, and 503 (72.5%) had neither FoB nor DS. | Cross-sectional nature of study.  Did not know whether women included in study who were categorized as having FoB and DS had received any treatment.  Unable to offer any support to women identified as having any problems as data were analyzed weeks later.  The tools were only for screening purposes and therefore could not be used for clinical diagnosis.  Findings cannot be generalized to the first and second trimester of pregnancy, as data were collected during the third trimester. |
| Massae et al., 2022  Pwani, Tanzania [66] | Longitudinal study  Health facilities | n = 694  Pregnant women | Wijma Delivery Expectancy/Experience Questionnaire versions A & B; Edinburgh antenatal and postnatal depressive scale | Prevalence rates of FoB and DS during pregnancy were 16% and 18.2%, respectively, and after childbirth, 13.9% and 8.5%. Some had FoB (6.4%) and DS (4.3%) at both timepoints. Obstetric complications decreased the odds of postpartum and persisting FoB (aOR 0.44, 95% CI 0.23–0.83). Caesarean section (aOR 2.01, 95% CI 1.11–3.65) and having more than 12 hours pass between admission and childbirth increased the odds of postpartum FoB (aOR 2.07, 95% CI 1.03–4.16). Postpartum DS was more common in women with an ill child/stillbirth/early neonatal death (aOR 4.78, 95% CI 2.29–9.95). Persisting DS was more common in single (aOR 2.59, 95% CI 1.02–6.59) and women without social support from parents (aOR 0.28, 95% 0.11–0.69). | Presence of interviewers influenced women's responses.  Not including high-risk women during recruitment, such as those who had experienced caesarean childbirth or did not expect vaginal birth. |
| Mbarak et al., 2019  Dar es Salaami, Tanzania [68] | Cross-sectional study  Hospital | n = 390  Women with pre-eclampsia and eclampsia | Face-to-face interviews | PPD was prevalent among 20.5% of women who had pre-eclampsia or eclampsia but varied with severity. Factors associated with PPD included young age (AOR = 10.13, 95% CI 1.99–52.02), being a single mother (AOR = 3.18, 95% CI 1.02–9.95), lower level of education (AOR = 3.83, 95% CI 1.45–10.16), having a perinatal death (AOR = 5.14, 95% CI 2.53–10.45), lack of family support (AOR = 7.06, 95% CI 1.25–39.90), and experience of stressful event during pregnancy (AOR = 15.14, 95% CI 2.38–96.19). | Cross-sectional design prevents causal inference.  Recall bias may have affected responses.  The single tertiary hospital setting limits the generalizability of the findings. |
| Mbatia et al., 2009  Dar es Salaam, Tanzania [14] | Cross-sectional study  Primary health facility | n = 14  10 nurses and 4 assistant medical officers (mean age 27, range 40–55 years) | Depression Attitude Questionnaire (DAQ), self-administered | Most respondents felt that rates of depression had increased in recent years, believed that life events were important in the etiology of depression, and generally held positive views about pharmacological and psychological treatments of depression, prognosis, and their own involvement in the treatment of depressed patients. | Limited by the small size of the sample of primary care workers.  All were working in Dar es Salaam — results may not be generalizable. |
| Mboya et al., 2020  Kilimanjaro, Tanzania [69] | Cross-sectional study  University | n = 402  Undergraduate students connected to the program in medicine and nursing | Self-reporting questionnaire | Residing off-campus (OR = 0.44, 95% CI 0.20–0.96) and perceived availability of social support (OR = 0.22, 95% CI 0.11–0.45) reduced the odds of mental distress. Students with family history of mental distress (OR = 2.60, 95% CI 1.04–6.57) and those with decreased grades than anticipated (OR = 3.61, 95% CI 1.91–6.83) had higher likelihood of mental distress. | Information bias whereby students might have misreported information related to different symptoms of mental distress as well as engagement in risk behaviors such as alcohol and drug use. |
| Mbwilo et al., 2010  Dar es Salaam, Tanzania [70] | Qualitative study  Community | n = 52  From 29 families caring for children and adolescents with mental disability | Semi-structured interviews; questionnaires | Major needs for children and adolescents are related to basic needs, education, health care, and security. Families need socioeconomic support. Families experienced discrimination and concerns for the future. | N/S |
| Messo, 2013  Dar es Salaam, Tanzania [71] | Cross-sectional study  Community | n = 100  Children (aged 9–15 years) who were survivors/involved in the Mbagala bomb blasts | Questionnaire; the Children's Revised Impact of Events Scale (CRIES-13) | 93% of children had PTSD symptoms. No statistically significant sex differences in PTSD symptoms. | Convenience sampling limits generalizability.  Reliance on self-reports is prone to bias.  CRIES-13 is a screening tool, not a full clinical diagnosis. |
| Mirza et al., 2006  Pemba, Zanzibar, Tanzania [72] | Cross-sectional study  Community | n = 600  General adult population from 300 households | Structured household questionnaire; Short Explanatory Model Interview (SEMI) | Help-seeking for CMD: 20.6%. First contact for CMD: Traditional healers (45%), PHC staff (36.2%). First contact for Epilepsy: PHC staff (50%), Traditional healers (30%). | Possible selection bias.  SEMI's cultural appropriateness and diagnostic range are limited.  Study did not measure true prevalence. |
| Mlaki et al., 2021  Kilimanjaro, Tanzania [73] | Secondary analysis of a cross-sectional study  Community | n = 296  Elderly adults aged ≥70 years in rural communities | Stage 1: Community Screening Instrument for Dementia (CSI-D) and CERAD 10-word test; Stage 2: 10/66 protocol using Geriatric Mental State (GMS)/AGECAT for depression diagnosis (DSM-IV criteria used for confirmation) | AOR (never married) = 2.77 (95% CI 1.36–5.66, p=0.01); AOR (good/very good health) = 0.22 (95% CI 0.10–0.47, p<0.001); AOR (moderate health) = 0.26 (95% CI 0.10–0.66, p=0.005). | Cross-sectional design (cannot infer causality).  Data originally for dementia (possible underestimation of mild depression).  Data collected 10 years earlier.  Limited generalizability. |
| Moledina et al., 2018  Dar es Salaam, Tanzania [74] | Cross-sectional study  Community | n = 384  Adults (≥18 years) from an Asian-migrant community | Structured questionnaire | OR for psychological stress/trauma = 6.37 (95% CI = 2.42–16.69); OR for family history = 2.57 (95% CI = 1.02–6.42); OR for sufficient income = 0.21 (95% CI = 0.06–0.77). | Possible under-representation (depressed individuals less likely to attend the community event).  Cross-sectional design (causality not inferable). |
| Munisi et al., 2022  Mwanza, Tanzania [75] | Cross-sectional study  Hospital | n = 376  Adults (18–85 years) with clinically diagnosed chronic kidney disease | Sociodemographic questionnaire; PHQ-9; self-administered | AOR 0.32 (95% CI 0.17–0.61, p<0.001) for inability to perform ADLs; AOR 0.56 (0.32–0.99, p=0.044) for 53–96 months since diagnosis; others non-significant. | Cross-sectional design (no causality).  Self-report and recall bias.  Hospital-based limits generalizability despite large catchment. |
| Mussa et al., 2023  Zanzibar, Tanzania [76] | Cross-sectional study  Hospital (Diabetic Clinic) | n = 267  Adults (≥18 years) diagnosed with diabetes for ≥3 months | Structured self-administered questionnaire; PHQ-9 (depressive symptoms) | Psychological distress (AOR=4.4; 95% CI 2.44–8.10; p<0.001); diabetic retinopathy (AOR=2.8; 95% CI 1.45–5.28; p=0.002); diabetic foot ulcer (AOR=0.1; 95% CI 0.04–0.49; p=0.003); impotence (AOR=0.4; 95% CI 0.20–0.68; p=0.002). High depression prevalence linked to poorer self-management, low adherence, and presence of complications (retinopathy). | Cross-sectional design (no causality).  Hospital-based sample (limits generalizability).  Exclusion of psychiatric diagnostic interviews (PHQ-9 only).  Self-report bias.  No assessment of medication or social support adherence beyond recall. |
| Mwita et al., 2021  Mwanza, Tanzania [78] | Cross-sectional study  Antenatal clinic, Medical Centre | n = 380  Pregnant women aged 20–47 years attending ANC | Structured self-administered questionnaire; Edinburgh Postnatal Depression Scale | Women whose partners were aged 31–40 years were statistically less likely to develop depressive symptoms (AOR 0.4, 95% CI: 0.2, 0.8, p=0.020). Those who were married (AOR 0.4, 95% CI: 0.2, 0.8, p=0.026). Polygamy type of marriage increased risk (AOR 9.8, 95% CI: 2.1, 45.2, p=0.003). College education protective (AOR 0.1, 95% CI: 0.0, 0.8, p=0.030). Partner support (AOR 0.1, 95% CI: 0.1, 0.6, p=0.008) and family support (AOR 0.2, 95% CI: 0.1, 0.9, p=0.041) were protective. Partner violence (AOR 7.1, 95% CI: 2.1, 23.4, p=0.001) increased risk. | Cross-sectional (cannot infer causality).  Self-reported symptoms leading to recall and reporting bias.  Single-hospital setting.  Cultural differences unassessed. |
| Mwita et al., 2024  Mwanza, Tanzania [79] | Cross-sectional study  Medical Centre | n = 386  Postpartum women within one week after delivery (aged 16–42 years) | Structured questionnaire; Edinburgh Postnatal Depression Scale (EPDS) for depression; Generalized Anxiety Disorder Scale (GAD-7) for anxiety | Depression: Married women less likely (AOR=0.7); polygamous marriage higher risk (AOR=3.7); caesarean delivery (AOR=1.9); complications (AOR=1.6); partner violence (AOR=5.4); low birth weight (<2.5 kg) protective (AOR=0.5).  Anxiety: Caesarean section (AOR=1.7); complications (AOR=1.4); partner violence (AOR=4.2); partner support protective (AOR=0.5). | Cross-sectional (no causality).  Self-report bias.  Interviewer presence may affect responses.  Hospital sample may not represent general population.  Possible underestimation due to social desirability bias. |
| Mwita et al., 2021  Mwanza, Tanzania [77] | Cross-sectional study  Antenatal clinic, Medical Centre | n = 380  Pregnant women aged 20–47 years | Questionnaire; Generalized Anxiety Disorder Scale (GAD-7, Swahili version) | College education (AOR=0.04, p=0.012); high income (AOR=0.2, p=0.013); planned pregnancy (AOR=0.6, p=0.030); partner support (AOR=0.4, p=0.013); partner violence (AOR=1.9, p=0.023). High prevalence of anxiety linked to socioeconomic and relational factors, strong association with IPV and low support. | Cross-sectional and recall bias due to self-report.  Hospital-based data.  Regional differences unaccounted for. |
| Myers et al., 2025 Arusha, Tanzania [80] | Mixed methods study — quantitative cross-sectional survey + qualitative semi-structured interviews | n = 71  Maasai women (community sample, ages 18–75) | Structured psychosocial surveys (Kessler-10 for psychological distress; Life Events Checklist for trauma exposure; voice-hearing screening questions modeled on Luhrmann et al. 2015) and semi-structured interview | Voice-hearers had higher distress (mean Kessler-10 = 29.1 vs. 25.8, p = 0.049) and more traumatic events (4.8 vs. 3.8, p = 0.073); logistic regression showed Kessler-10 score predicted AVH (AOR = 1.09, p = 0.042). Voice-hearing associated with high distress, trauma, and social adversity; absence of care likely worsens psychosocial burden; low clinical service utilization due to stigma and geographic inaccessibility.  Themes: Extreme food and water insecurity; drought; livestock loss; domestic violence; child mortality; poverty; gender inequality; religious framing of voices (Satan, Holy Spirit, dead relatives); "thinking too much" and "depression" (engilishoto) as local idioms. | Data collected only during dry season.  Translation bias possible.  Limited psychometric validation of tools in Maa. |
| Ndosi et al., 2002  Dar es Salaam, Tanzania [82] | Prospective clinical study  Hospital | n = 86  Postpartum women diagnosed with puerperal psychosis (within six weeks postpartum) | Questionnaire; mental status examination; physical examination | 60 mothers (69.8%) developed psychosis within one week after delivery, 18 (20.9%) within a fortnight, 5 (5.8%) within three weeks, and 3 (3.5%) within six weeks postpartum. Most mothers received social support from their extended families. Organic psychosis was found in four-fifths of the mothers and schizophrenia in 8.1%. A high rate of early onset puerperal psychosis (3.2/1,000 births), predominantly in young primiparous women, was found. | Hospital-based data; small sample size; absence of control group; diagnostic bias (limited lab screening); cultural and socioeconomic variables not deeply analyzed; exclusion of untreated home cases. |
| Ndosi et al., 1997  Dar es Salaam, Tanzania [81] | Descriptive, observational study  Hospital (Medical Centre) | n = 300  Suicide attempters (13–60 years) admitted to hospital | Semi-structured interview; questionnaire | 80.7% triggered by social conflicts; 39% had mental/physical disorders (psychosis 12.3%, major depression 9%, drug abuse 4%); 65% of overdoses involved chloroquine; 40% demonstrative intent, 31% genuine death wish; 18.3% family history of mental illness. | Hospital-based, underreporting due to stigma.  Limited psychiatric diagnostic resources.  Potential recall bias. |
| Ngocho et al., 2022  Kilimanjaro, Tanzania [83] | Cross-sectional study  Primary health centers | n = 1,039  Pregnant women attending their first antenatal care | Questionnaire; Edinburgh Postnatal Depression Scale (EPDS); Perceived Availability of Support Scale; Partner Support Scale; WHO Short Intimate Partner Violence Tool | Probable depression was more prevalent among women who were not married compared with married women (PrR = 2.1, 95% CI 1.5, 3.0), women who were living with HIV compared with women who were not living with HIV or had unknown HIV status (PrR = 2.2, 95% CI 1.2, 4.1), and women who had a lifetime history of violence (PrR = 4.3, 95% CI 2.9, 6.2). Probable depression was less prevalent among women who had informal income generating activities (PrR = 0.7, 95% CI 0.5, 1.0), women who reported more general social support (PrR = 0.93, 95% CI 0.90, 0.95), and women who reported more partner support (PrR = 0.85, 95% CI 0.82, 0.89). In the final multivariable log-binomial regression analysis, three factors (reported violence, marital status, and partner support) were associated with probable antenatal depression. | EPDS is a screening rather than diagnostic tool.  Exclusion of women under the age of 18.  Does not include an assessment of antenatal depression in this vulnerable population.  39% of participants unaware of HIV status. |
| Ngoma et al., 2003  Dar es Salaam, Tanzania [84] | Cross-sectional study  Primary health clinics and traditional healer centers | n = 899  General adult population aged 15–59 years | Questionnaire for socio-demographics; Short Explanatory Model Interview (SEMI); Clinical Interview Schedule — Revised (CIS–R) | The prevalence of common mental disorders among traditional healing center (THC) patients (48%) was double that of primary health care patients (24%). | Findings may have limited generalizability because it was confined to Dar es Salaam with a small, non-representative sample of two primary health centers and eight traditional healers, introducing potential biases that could account for the observed differences in common mental disorder prevalence between settings. |
| Njiro et al., 2021  Dar es Salaam, Tanzania [85] | Cross-sectional study  Community-based, within police stations and special police units | n = 550  Police officers | Questionnaire for sociodemographic information; Patient Health Questionnaire-9 (PHQ-9); Interpersonal Support Evaluation List-12 | Adjusted OR for low social support and depression = 28.04 (95% CI 8.42–93.37); for suicidality = 10.85 (95% CI 3.56–33.08). | Self-administered questionnaire led to missing data.  Potential social desirability bias. |
| Nordgreen & Havik, 2012,Manyara, Tanzania [86] | Qualitative analysis of clinical data  Hospital | n = 10  Patients with panic disorder (PD) | Interview — Structured Clinical Interview for the Diagnostic and Statistical Manual of Mental Disorders, Fourth Edition, DSM-IV (SCID) | Five patients, who explained their symptoms as a consequence of physical disease and/or reaction to stressful life events, also saw their symptoms as caused by "spells" or "bad eye" (coercion), most likely brought on by a neighboring clan. These patients had had their spell diagnosed by a traditional healer and symptoms of a spell included ill health, lack of concentration, poor school results, and social problems. | The PD diagnosis has relevance to a rural Tanzanian setting, and that brief CBT interventions were applicable in this setting. |
| Nyundo et al., 2020  Dar es Salaam and Dodoma, Tanzania plus other Sub-Saharan Africa [87] | Cross-sectional study  Community | N/A  Adolescents aged 10–19 years | Survey | The prevalence of suicidal behavior over the last 12 months ranged between 1.2% and 12.4% in the eight sites. Depressive symptoms and suicidal ideation/behaviors were associated with older age, female sex, food insecurity, poor access to health care, and substance use. | Cross-sectional design prevents causality. |
| Patil & Hadley, 2008  Tanzania [88] | Short report  Community | n = 408  Mothers | Interviews with mothers using a measure of psychological distress | There were no differences in anxiety, depression, or the combined measures of distress across the measures of marriage types. | Limited power to detect relatively small effect sizes between groups — due in part to small sample sizes in the polygynous groups.  The measures of psychosocial stress may be inappropriate for the study populations. |
| Pauley et al., 2025 Kilimanjaro, Tanzania [89] | Cross-sectional, mixed-methods study | Not stated (NS)  General population | (1) PHQ-9; (2) AUDIT; (3) Drinkers' Inventory of Consequences (DrInC); (4) basic demographic data; (5) self-reported alcohol use data; plus a semi-structured interview | ED women held the highest prevalence of major depressive disorder (25%) compared to RHC women (11%) and ED men (7.9%) (p < 0.001). Depressive symptoms were associated with higher AUDIT scores for ED men (R2 = 0.11, p < 0.001). Qualitative analysis showed that while present for women, social support networks were notably absent for men, playing a role in alcohol use. For men, alcohol was described as a coping mechanism for stress. The intersectionality of gender, alcohol use, and depression is influenced by sociocultural and behavioral norms in Moshi. Increased pressure placed on men to be the financial caretakers for their families; social norms mediate that in the face of this stress, men are expected to "keep problems to themselves as a sign of strength" and "find solutions all by yourself," while women in the community are encouraged to talk to other women. | Depression and alcohol use questions were added midway through data collection, though thematic saturation was achieved using rigorous qualitative procedures.  Key drivers of MDD, such as past trauma, medical conditions, and genetic factors, were not measured in the quantitative survey, limiting explanatory depth. |
| Pike & Patil, 2006  Manyara, Tanzania [88] | Qualitative study  Community | n = 113  Women (49 Datoga women and 64 Iraqw women) | Culturally specific questionnaire (mental health assessment instrument modified from the Swahili version of the HSCL-25) and focus group interviews | Both groups of women identified hunger, the lack of animals (particularly cattle), and health/illnesses as the most common major stressors. Other frequently cited stressors included crop failure, general fears of violence, paying taxes, and no money for basic needs. Datoga women express and/or experience greater burdens of anxiety. | Biased responses toward over-reporting due to the organization of the interview.  Cross-sectional nature of the survey. |
| Prencipe et al., 2021  Iringa and Mbeya, Tanzania [91] | Cross-sectional study  Community | n = 2,458  Adolescents (aged 14–19) | Survey | Factors associated with depressive symptoms: experiencing five or more household economic shocks (β=2.40; 95% CI 1.48 to 3.32); experiencing droughts/floods (β=0.76; 95% CI 0.36 to 1.17); being in a relationship (β=1.82; 95% CI 1.30 to 2.33); and having moderate (β=1.26; 95% CI 0.80 to 1.71) or low social support (β=2.27; 95% CI 1.81 to 2.74). Exclusive schooling was protective compared with being engaged in both school and paid work (β=1.07; 95% CI 0.05 to 2.61) and not engaged in either (β=0.73; 95% CI 0.24 to 1.22). Household size and relationship status were more important factors for girls, while employment status and extreme precipitation were more important for boys. | Cross-sectional design prevents causal inferences especially for time-varying factors such as employment and relationship status.  The homogeneously poor sample may underestimate neighborhood and economic associations. |
| Ramos de Oliveira et al., 2022, Morogoro, Tanzania [92] | Cross-sectional study  Community | n = 981  Women (mothers) and their children aged 18 to 36 months | Structured interviews; Tanzania Demographic and Health Survey; Maternal Depressive Scale — PHQ-9; Caregiver-Reported Early Childhood Development Instruments (CREDI); UNICEF Multiple Indicator Cluster Surveys (MICS) | Physical intimate partner violence reduced scores by −0.022, and physical and sexual intimate partner violence by −0.045. Reduced child socioemotional scores by −0.073. No significant impact after adjusting for intimate partner violence and maternal depression. | Limited generalizability to other regions.  Reliance on non-diagnostic tools for depressive symptoms.  Exclusion of psychological and financial intimate partner violence.  Focusing on quantity rather than quality of child stimulation.  Limited measures for harsh discipline.  Excluded other caregivers, such as fathers.  Faced a risk of false-positive findings in secondary analyses. |
| Rogathi et al., 2017  Kilimanjaro, Tanzania [93] | Prospective cohort study  Antenatal clinics | n = 1,013  Pregnant women (aged 18 years and above) | Interviews; Edinburgh Postpartum Depression Scale (EPDS); WHO IPV Questionnaire; health records, cross-checked for HIV and health status; gestational age was confirmed with ultrasound | 12% of women had postpartum depression (EPDS score ≥13).  IPV during pregnancy: 30% experienced IPV — emotional (22.3%), sexual (15.7%), physical (6.2%).  Odds of Postpartum Depression: Any IPV: 3 times higher odds (AOR = 3.10). Physical violence: 2 times higher odds (AOR = 2.15). Sexual violence: Nearly 2 times higher odds (AOR = 1.98).  Age-Specific Risk: Women aged 18–24 exposed to physical violence had 4 times higher odds of postpartum depression. | The Edinburgh Postpartum Depression Scale was a screening tool, not a diagnostic tool, and no psychiatric validation was done.  Intimate partner violence may have been underreported due to stigma or fear.  Self-reported data on IPV and depression may have recall bias.  The study did not include experiences of violence or depression before pregnancy.  The follow-up ended at 40 days postpartum, missing long-term effects.  Findings may not apply to other regions or cultures. |
| Rosario et al., 2017  Mwanza, Tanzania [94] | Qualitative, descriptive phenomenological study  Antenatal care clinics | n = 10  Pregnant and postpartum women participants | Semi-structured interviews | Anxiety about fetal/infant health, maternal health, childbirth, and parenting ability.  Limited understanding of pregnancy symptoms and inadequate guidance from healthcare providers.  Emotional and financial neglect, partner absence, and conflicts increased anxiety.  Poor communication, lack of empathy, and financial barriers exacerbated worries.  Concerns about infection, transmission to the baby, and medication side effects.  Prayer and faith provide emotional relief and hope.  Gender inequality, poverty, and stigma worsened anxiety. | The exclusion criterion was amended to include early postpartum women as many women from the associated quantitative study began to deliver before recruitment had been completed.  The stigma of mental illness and presence of a local third party (translator) may have affected the level of participation, engagement, or willingness of women to participate in the interviews.  Due to time constraints, it was not feasible to return to the participants to present initial findings and collect feedback, as per Colaizzi's method. |
| Rwakarema et al., 2015  Mwanza, Tanzania [95] | Cross-sectional study  Antenatal care services | n = 397  Pregnant women attending antenatal care services | Edinburgh Postnatal Depression Scale (EPDS); face-to-face interviews | Pregnancy-Related Anxiety: OR 1.36. Poor Partner Relationships and Low SES: OR 82.34. Limited Family Support and Decision-Making also increased risk. Trimester Distribution: Depression rates — 1st trimester (15.7%), 2nd trimester (50.0%), 3rd trimester (34.3%). | Cross-sectional design cannot establish causality.  Facility-based sampling excludes women not accessing antenatal care.  Social desirability and recall bias may affect responses.  Did not assess intimate partner violence or HIV status.  Swahili version not validated; it is a screening, not a diagnostic tool.  Some categories had small sample sizes, leading to wide confidence intervals. |
| Rweyemamu et al., 2024, Dodoma, Tanzania [96] | Cross-sectional study  Community | n = 224  Undergraduate students | Modified version of the SRQ-20 (Self-Reporting Questionnaire); captured knowledge, self-reported symptoms, and factors contributing to mental distress | Most recognized symptoms like depression (74.6%) and anxiety (68.8%). Alcohol consumption (aPR = 1.61); marital status (higher in married students); year of study (higher in 3rd/4th years); financial difficulties (86.2%); sexual relationships (79.0%); low grades (62.1%). | Cross-sectional design limits causal relationships.  Small sample size limits generalizability and statistical power.  Mental distress was self-reported, increasing risks of desirability bias and recall bias.  Focused only on undergraduate students, excluding other groups within the university.  Did not assess other potential contributors to mental distress (e.g., trauma, social support). |
| Saadi et al., 2018  Dar es Salaam, Tanzania [97] | Prospective cohort study  Hospital | n = 76  Adults (≥18 years) with ischemic or hemorrhagic stroke | Modified Rankin Scale (mRS); Berkman-Syme Social Network Index (SNI); PHQ-9 | 65% of participants had high social connectedness (SNI = 3 or 4). 13% reported low social connectedness (SNI = 0 or 1), indicating social isolation.  Higher social network scores were associated with fewer depressive symptoms (p < 0.0001) and lower disability (p = 0.061).  Higher depressive symptoms were moderately correlated with greater disability (r = 0.52, p < 0.0001). | Small sample size limits statistical power.  High mortality and loss to follow-up may bias results.  PHQ-9 and SNI may not fully capture the cultural context of Tanzania.  Reliance on proxies may introduce bias in reported outcomes.  The Berkman-Syme SNI may not fully reflect Tanzanian social networks.  No data on pre-stroke depression or social networks to establish baseline comparisons. |
| Sajatovic et al., 2020  Dar es Salaam, Tanzania [98] | Cross-sectional study  Hospital | n = 100  Adults aged 18+ diagnosed with schizophrenia or schizoaffective disorder | Tablets Routine Questionnaire (TRQ); Drug Attitudes Inventory (DAI); Psychiatric Rating Scale (BPRS); Clinical Global Impressions Scale (CGI); AUDIT; ASSIST | 10% had alcohol dependence; 33% had substance use issues. Worse adherence linked to inpatient status, severe symptoms, poor medication attitudes, and alcohol use. | Cross-sectional design limits ability to infer causality.  Focused only on poorly adherent patients, limiting generalizability to the broader CPD population.  Did not assess barriers like poverty, clinical access, and patient-clinician communication.  Small sample size limits power to detect nuanced associations.  No control for multiple comparisons, and gender differences were not fully explored. |
| Saleem et al., 2023  Dar es Salaam, Tanzania [99] | Cross-sectional study  Community | n = 200  Women aged 18+ who use drugs (specifically heroin) | Structured face-to-face interviews; PHQ-9; GAD-7; Modified SU-SMS (Substance Use Stigma Mechanism Scale) | 67.5% of participants reported symptoms of depression (PHQ-9 scores > 9). 43.7% reported symptoms of anxiety (GAD-7 scores > 9). Internalized drug use stigma was significantly associated with depression (AOR = 1.34). Enacted stigma from health workers (AOR = 2.02) and anticipated stigma from family (AOR = 1.49) were associated with anxiety. Participants with prior attempts to stop heroin use were nearly three times more likely to report depressive symptoms (AOR = 2.99). 61.9% experienced physical violence, and 35.8% experienced sexual violence in the past year. | Non-random sample — results not generalizable.  Selection bias — potential oversampling of women with severe drug use or trauma.  Unmeasured confounders — limited data on healthcare engagement and stigma sources.  Small subsample for intersectional analysis — limited ability to assess combined drug use and HIV stigma. |
| Sariah et al., 2014  Dar es Salaam, Tanzania [100] | Qualitative study  Hospital | n = 14  Adults diagnosed with schizophrenia (per DSM-IV criteria) | In-depth interviews covering personal and environmental risk and protective factors for relapse; audio-recorded interviews transcribed and analyzed using NVivo 9 software | People with schizophrenia and their caregivers (all of whom were relatives) perceived nonadherence to antipsychotic medication as a leading risk factor of relapses; other risks included poor family support, stressful life events, and substance use. Family support, adherence to antipsychotic medication, employment, and religion were viewed as protective factors. | Recruiting caregivers who had accompanied patients to the Psychiatric Out-patient Department probably contributed to inclusion of caregivers with very good relationships with their patients and interest in patient care.  They might have also given socially desirable answers.  The small sample may limit the transferability of the findings — particularly in relation to ethnicity and social economics conditions. |
| Uriyo et al., 2013  Kilimanjaro, Tanzania [101] | Cross-sectional study  Community | n = 1,922  Mothers (15–48 years old) for children 0–36 months | Survey; Shona Symptom Questionnaire (SSQ) | Risk of CMD was associated with verbal abuse, physical abuse, a partner who did not help with the care of the child, being in a polygamous relationship, a partner with low levels of education, and a partner who smoked cigarettes. Cohabiting appeared to be protective. | Refusal rate of working mothers in the urban areas to participate in the study.  Self-reported HIV could give an underestimation.  Cross-sectional design.  Response bias due to the sensitive nature of reporting physical abuse in this culture. |
| Wall et al., 2018  Mwanza, Tanzania [102] | Secondary analysis, survey, cross-sectional  Community | n = 212  Pregnant women over 18 years | Secondary dataset | Approximately three-quarters of participants (78.2%) were multiparous; most participants were in a relationship (89.6%) and over three-quarters of participants (77.8%) identified that they were married. Although nearly all participants (97.2%) had some level of formal education, most had only completed primary school (64.6%). Socioeconomic status was higher than expected with 57.1% of participants scoring 12 or better (out of 18) on the SEW-Q and 9.0% scoring 5 or less. PRA-Q scores ranged from 0 to 24 with a median score of 10 (IQR=8–13). When PRA was dichotomized as 'high' and 'low-moderate' categories, 6.1% of participants (n=13) had high anxiety. | Secondary data, sample size, and tools not developed for Tanzania. |
| Gao et al., 2025, Tanzania, Zanzibar [104] | Cross-sectional study –  Hospital | 330 infertile women | Survey, PHQ-9 and GAD-7 | Factors correlated with high depression level: age>35 years and low education level, For high anxiety level: low education level | Use of different tools in comparison with other studies. The site and design may result in selection bias, prevalence over estimation. |
| Nkuba et al., 2018, Tanzania [105] | Cross-sectional study –  Secondary schools | 700-secondary school students and 333 parents/primary caregivers | Questionnaires, including the Strengths and Difficulties Questionnaire (SDQ) and the Conflict Tactics Scale (CTS) | 41% of students reported elevated mental health problems. 31% of parents reported elevated levels of mental  health problems of their children | The study is cross-sectional, so causality cannot be inferred. Data are based on self-reports. |
| Wesselhoeft et al., 2020,  Denmark, Vietnam and Tanzania [106] | Cross-sectional study-  Community | 4516: Women who had given birth (From Denmark 2069, Vietnam 1278 and Tanzania 1169) | Edinburgh Postnatal Depression Scale and self-reported demographics. | Women from Tanzania and Denmark were more likely to have an EPDS total score above cut-off 12 (12.6% and 6.4%). A low level of education was associated with significantly more depressive symptoms after adjusting for country (p<0.001) | Cross-sectional design, Edinburgh Postnatal Depression scale developed outside Vietnam, Tanzania (and Denmark). |
