# Supplementary material for: Structural and health system determinants of mental health in Tanzania: mapping policy recommendations to the WHO comprehensive mental health action plan 2013–2030
Source: Front Public Health. 2026 Jul 10;14:1878653. doi: 10.3389/fpubh.2026.1878653 (PMC13398205; doi:10.3389/fpubh.2026.1878653)
Supplement: Supplementary file 2 [file Supplementary_file_2.docx]

**Supplementary Table 2. Policy Recommendations Categorized by WHO Comprehensive Mental Health Action Plan (2013–2030) Objectives**

| **Author / Year / Region** | **Leadership and Governance** | **Service Integration and Expansion** | **Workforce Development** | **Access and Service Quality** | **Medication Availability** | **Promotion and Prevention** | **Information Systems, Evidence, and Research** |
| --- | --- | --- | --- | --- | --- | --- | --- |
| Adams et al., 2021  Kilimanjaro, Tanzania [31] |  | Integrate mental health care into primary care services |  |  |  | Awareness programs to improve recognition and reduce stigma.  Strengthen social support systems (family, religious, community groups). |  |
| Alemu et al., 2020  Zanzibar, Tanzania [32] |  |  |  |  |  | Integrate women's social networks into prevention and management after maternal complications.  Pay special attention to women with intercurrent illness or perinatal loss, as they are at higher risk. |  |
| Ambikile, 2023  Dar es Salaam, Tanzania [34] |  |  | Strengthening education to provide structured mental health education for caregivers, communities, and decision-makers to reduce stigma | Improve services to fund outreach/home visits and establish rehabilitation centers to support recovery.  Foster collaboration and engage traditional and religious healers to improve referrals and reduce treatment delays. |  |  |  |
| Blixen, 2020  Dar es Salaam, Tanzania [38] |  |  |  | Stronger services |  | Develop educational materials for patients, caregivers, and communities to counter misconceptions and improve knowledge.  Anti-stigma action.  Culturally adapted adherence interventions. |  |
| Bondestam et al., 1990  Zanzibar, Tanzania [39] |  | Engage traditional healers | Training staff |  |  | Educate communities | Improve primary healthcare detection |
| Cherewick et al., 2024  Dar es Salaam, Tanzania [40] |  |  |  |  |  | Introduce early, skills-based interventions.  Support mastery and communal coping approaches. |  |
| Decaro et al., 2016  Mwanza, Tanzania [42] |  | Integrating culturally sensitive approaches to address stigma in maternal and child health care services |  |  |  |  |  |
| Gao et al., 2025  Zanzibar, Tanzania [104] |  |  |  |  |  | Community education is recommended as a part of the policy of mental health services. |  |
| Herlosky et al., 2020  Arusha, Tanzania [44] |  | Integrating psychological health screening among infertile patients using Swahili-translated PHQ-2/GAD-2 |  |  |  |  |  |
| Hill et al., 2020  Dar es Salaam, Tanzania [45] |  | Integrate using EPDS in combination with qualitative methods in evaluating postpartum health |  |  |  |  |  |
| Hill et al., 2017  Dar es Salaam, Tanzania |  | Lay counselor and social support models should be tested |  |  |  |  |  |
| Holm-Larsen et al., 2019  Kiliamanjaro, Tanzania |  | Behavioral interventions to be implemented |  |  |  |  |  |
| Hovland et al., 2025  Tanga, Tanzania [20] |  |  |  |  |  | Screening for depression in pregnant women reporting intimate partner violence |  |
| Howorth et al., 2019  Kilimanjaro, Tanzania [46] |  |  |  |  |  |  | Understanding culturally specific concepts of depression may allow more accurate diagnosis, improve service use and availability, and reduce stigma |
| Iseselo et al., 2020  Dar es Salaam, Tanzania [49] |  |  |  |  |  | Advocating mental health awareness for caregivers and community members |  |
| Iseselo et al., 2017 [48]  Dar es Salaam, Tanzania |  |  |  |  | Ensure adequate and regular supply of psychotropic medications for mental health |  |  |
| Iseselo et al., 2016  Dar es Salaam, Tanzania [47] | Collaborative approach between health care providers and the government to address the needs of the caregiver and the family in general |  |  |  |  |  |  |
| Ivanova et al., 2022  Mbeya and Songwe, Tanzania [50] | Implementation of health policies and strategies aiming to improve health and well-being of the local population |  |  |  |  |  |  |
| Kaaya et al., 2010  Dar es Salaam, Tanzania [54] |  | Integrating screening for depression in routine antenatal care |  |  |  |  |  |
| Knettel et al., 2018  Arusha and Kilimanjaro, Tanzania [56] |  | Improve integration across settings.  Expand school-based counseling.  Engage traditional/religious healers | Increase resources and training |  |  | Reduce stigma via public education |  |
| Lugata et al., 2021  Kilimanjaro, Tanzania [60] |  |  |  | Better mental health support services in higher education institutions |  |  |  |
| Magnusson et al., 2021  Kilimanjaro, Tanzania [61] |  |  |  |  |  |  | Need for validated resilience tools in Tanzanian context |
| Mahenge et al., 2013  Dar es Salaam, Tanzania [63] |  | Integrate mental health screening into antenatal care | Training and education of antenatal care providers |  |  |  |  |
| Mahenge et al., 2015  Dar es Salaam, Tanzania [64] |  |  | Knowledge and skills for recognition and management of mental health disorders need to be incorporated in the in-service training course curricula of doctors and other healthcare professionals |  |  |  |  |
| Mahenge et al., 2018 [62]  Dar es Salaam, Tanzania |  |  |  |  |  | Screen and respond at an early level for women who have suffered adverse childhood experiences, IPV during pregnancy, and postnatal depression in postnatal clinics |  |
| Manongi et al., 2020  Kilimanjaro, Tanzania [65] |  | Screening for depression and IPV experiences among pregnant women as part of the routine antenatal services |  | Offering supportive counseling and/or referral to appropriate services |  | Formation of formal community social support |  |
| Mbatia et al., 2009  Dar es Salaam, Tanzania [14] |  |  | Strengthen the training and continuing professional development of primary health care workers in the detection and management of depression |  |  |  |  |
| Mboya et al., 2020  Kilimanjaro, Tanzania [69] |  | Establishing student drop-in centers that will provide counselling services to students experiencing different mental health issues |  |  |  | Awareness creation as well as counselling to help students with mental health.  Creating social and recreational activities. |  |
| Mbwilo et al., 2010  Dar es Salaam, Tanzania [70] |  | "Patient"-oriented, community/family-based health services to support management of chronic or life-long conditions.  Provision of guidance and supervision to the families.  Community and family/home-based care |  |  |  | Improve support of caregivers to children with mental disabilities |  |
| Messo, 2013  Dar es Salaam, Tanzania [71] | The government and health sector should be prepared for the psychological consequences of future disasters |  |  |  |  |  |  |
| Mirza et al., 2006  Pemba, Zanzibar [72] |  | Integrate a basic mental health care package into PHC clinics.  Collaborate with traditional/religious healers |  |  |  | Improve public and health staff knowledge/attitudes |  |
| Mlaki et al., 2021  Kilimanjaro, Tanzania [73] |  | Develop effective community-based interventions to reduce treatment gap |  |  |  |  |  |
| Moledina et al., 2018  Dar es Salaam, Tanzania [74] |  | Integrating regular screening in the community to the mental health programs |  |  |  |  |  |
| Munisi et al., 2022  Mwanza, Tanzania [75] |  |  | Training psychiatrists and counselors |  |  | Early screening for depression |  |
| Njiro et al., 2021  Dar es Salaam, Tanzania [85] |  |  |  |  |  | Screening of mental health symptoms including depression and suicidality.  Mental health awareness education.  Offering early intervention mental health services are required in the police force. |  |
| Nkuba et al., 2018  Tanzania [105] |  |  |  |  |  | Violence prevention and child protection programs in schools |  |
| Nordgreen & Havik, 2012  Arusha, Tanzania [86] |  |  |  | Adapted CBT interventions for panic disorder can be used in rural clinical settings |  |  |  |
| Pike & Patil, 2006  Arusha,Tanzania [88] |  |  |  |  |  |  | Culturally sensitive approach is needed for mental health assessments and interventions |
| Rwakarema et al., 2015  Mwanza, Tanzania [95] | Tackle stigma.  Strengthen healthcare infrastructure | Integration of screening and counseling to antenatal care | Training providers | Improve access |  |  |  |
| Rweyemamu et al., 2024  Dodoma, Tanzania [96] |  | Integrate depression screening and counseling to antenatal care | Training staff |  |  |  | Ensure Swahili EPDS is reliable for local use |
| Saadi et al., 2018  Dar es Salaam, Tanzania [97] |  |  |  | Improve follow-up |  |  | Validate tools like PHQ-9 for Tanzania |
| Simon et al., 2021  Dar es Salaam, Tanzania [103] |  | Screening for alcohol use |  |  |  |  |  |
| Wall et al., 2018  Mwanza, Tanzania [102] |  |  |  |  |  | Development of pregnancy-related anxiety prevention strategies |  |
| Wesselhoeft et al., 2020  Denmark, Vietnam, and Tanzania [106] |  | A nurse-delivered mental health intervention |  |  |  |  |  |
