# Supplementary material for: Structural and health system determinants of mental health in Tanzania: mapping policy recommendations to the WHO comprehensive mental health action plan 2013–2030
Source: Front Public Health. 2026 Jul 10;14:1878653. doi: 10.3389/fpubh.2026.1878653 (PMC13398205; doi:10.3389/fpubh.2026.1878653)
Supplement: Supplementary file 3 [file Supplementary_file_3.doc]

**Supplementary Table 3. Relationship between reported determinants and policy recommendations**

| **Reported determinant or barrier** | **Most relevant recommendation domains** | **Relationship observed in the review** |
| --- | --- | --- |
| Poverty, unemployment, and financial barriers | Leadership and governance; access and service quality; financing-related policy action | Frequently reported as determinants, but less often translated into explicit governance, financing, or social protection recommendations. |
| Food insecurity and other material deprivation | Promotion and prevention; leadership and governance; intersectoral action | Frequently reported as structural factors but rarely linked to concrete intersectoral policy recommendations. |
| Workforce shortages and limited provider capacity | Workforce development; service integration and expansion | More directly reflected in recommendations on training, task-sharing, and integration into primary care or other existing services. |
| Medication shortages, cost, and limited availability | Medication availability; service integration and expansion | Repeatedly identified as barriers, but only one study explicitly recommended improving psychotropic medicine supply. |
| Lack of routine screening and culturally adapted tools | Information systems, evidence, and research; promotion and prevention; service integration | Reflected in recommendations on screening, early identification, and validation of locally appropriate tools, but still less frequent than service integration recommendations. |
| Geographic access barriers, weak referral pathways, and limited follow-up | Access and service quality; service integration and expansion | Commonly reflected in recommendations on referral pathways, outreach, rehabilitation, follow-up care, and community-based services. |

Note: The table is based on the categories reported in the manuscript.
